# Diurnal patterns of accelerometer-measured physical activity and sleep and risk of all-cause mortality: a follow-up of the National Health and Nutrition Examination Surveys (NHANES)

**DOI:** 10.1186/s12966-024-01673-9

**Published:** 2024-10-18

**Authors:** Yue Zhang, Mika Kivimäki, Rodrigo M. Carrillo-Larco, Yangyang Cheng, Yaguan Zhou, Hui Wang, Changzheng Yuan, Xiaolin Xu

**Affiliations:** 1 School of Public Health, The Second Affiliated Hospital, Zhejiang University School of Medicine, Hangzhou, 310058 Zhejiang China; 2 The Key Laboratory of Intelligent Preventive Medicine of Zhejiang Province, Hangzhou, China; 3 UCL Brain Sciences, University College London, London, UK; 4 Emory Global Diabetes Research Center, Emory University, Atlanta, GA USA; 5 Hubert Department of Global Health, Rollins School of Public Health, Emory University, Atlanta, GA USA; 6 Department of Nutrition, Harvard T.H. Chan School of Public Health, Boston, MA USA; 7 School of Public Health, Faculty of Medicine, The University of Queensland, Brisbane, Australia

**Keywords:** Diurnal patterns, Physical activity, Sleep, Time of lifestyle behaviour, All-cause mortality

## Abstract

**Background:**

Physical activity and sleep are established modifiable lifestyle factors, but the optimal time of the day of these behaviours for health is unknown. This study examined the independent and joint associations of diurnal patterns of physical activity and sleep with all-cause mortality.

**Methods:**

This prospective cohort study included 6,673 participants who have attended the accelerometer assessment in the 2011–2014 National Health and Nutrition Examination Surveys (NHANES). Diurnal patterns of accelerometer-measured physical activity and sleep were identified using K-means clustering analysis. All-cause mortality was ascertained from the accelerometer measurement to December 31, 2019 (median follow-up 6.8 years). Survey-weighted Cox proportional hazard models were performed to estimate the independent and joint associations of diurnal patterns of physical activity and sleep with all-cause mortality.

**Results:**

Diurnal patterns identified were: early-morning (32.4%), midday (42.5%), and late-afternoon (25.1%) for physical activity; and irregular sleep (37.4%), morning lark (33.6%), and night owl (29.0%) for sleep. After adjusting for volume of physical activity, sleep duration and other potential covariates, the early-morning physical activity pattern (hazard ratio 1.36, 95% confidence interval 1.13–1.64) and irregular sleep pattern (1.42, 1.01–1.99) were independently associated with higher risk of all-cause mortality, compared with midday physical activity and morning lark sleep patterns, respectively. In addition, participants with the combined pattern of early-morning physical activity and irregular sleep had higher risk of all-cause mortality compared to those with midday physical activity combined with a morning lark sleep pattern (1.92, 1.33–2.78). Several sociodemographic differences were observed in the strength of these associations.

**Conclusions:**

Wearable activity-rest monitoring data showed that peak physical activity in the early morning and irregular sleep diurnal patterns are associated with increased mortality risk, and the combination of these patterns further exaggerated the risk. Public health program should acknowledge that the diurnal patterns of physical activity and sleep, in addition to their duration and frequency, may play a crucial role in lifestyle-based health promotion and management strategies.

**Supplementary Information:**

The online version contains supplementary material available at 10.1186/s12966-024-01673-9.

## Background

Adhering to a healthy lifestyle, such as engaging in habitual physical activity (PA) and maintaining optimal sleep duration, is the cornerstone and cost-effective strategy for the primordial, primary, and secondary prevention of non-communicable diseases throughout the life span [1, 2]. There is strong evidence supporting the protective effects of regular PA and adequate sleep duration against a multitude of adverse health consequences (e.g., cardiovascular diseases [CVD], diabetes, dementia) and premature mortality [3, 4]. Accordingly, the American Heart Association has proposed *Life’s Essential 8* which included participation in PA and healthy sleep as key targets for reducing the risk of major health problems [5]. While the benefits of higher volume and intensity of PA and appropriate sleep duration are well documented, less is known about whether the time of day we do physical activity and sleep affects health outcomes.

Given the consensus on the significant role of circadian rhythms in physiology and diseases [6], examining the impact of diurnal patterns of lifestyle behaviours on health is warranted. To date, the results on the association of time patterns of PA and sleep with health outcomes are inconsistent [7–18]. Using data from UK Biobank, some studies found that early-morning exercise may be associated with higher risks of all-cause and CVD mortality [8, 9], while contrasting findings suggested morning PA could aid in reducing the risks of CVD and diabetes [11, 13]. Conflicting results also exist in several randomized trail studies. One study based on 11 men with type 2 diabetes (T2D) showed that exercise in the morning was less efficacious than exercise in the afternoon in lowing blood glucose [7]; another randomized crossover trail study found morning exercise may benefit the management of glycaemia in patients with T2D [12]. For sleep patterns, some studies reported that individuals who are night owls (i.e., being active late into night and sleeping until relatively late in the day) may have higher risks of metabolic dysfunction and CVD [14–16], while others have suggested sleep regularity may be a stronger predictor of mortality risk [17, 18]. The measurement methods of sleep varied across these studies, with sleep chronotype being measured by self-reported questionnaires [15, 16], and sleep regularity being assessed by accelerometers [17, 18]. Considering these mixed results, further large-scale studies based on different populations with accelerometer-measured data are necessary to achieve more conclusive results.

Beyond their independent health effects, PA and sleep duration have been hypothesized to be interrelated. It is suggested, for instance, that more PA could help maintain a better sleep quality [19], while prolonged sleep duration might reduce the time for PA [20] among middle- or older-aged adults. The observed joint associations of PA and sleep with cognitive decline and all-cause, CVD and cancer mortality [21, 22] support this hypothesis. However, these studies focused only on the volume and intensity of PA and the duration of sleep. To date, we found no studies have examined the diurnal patterns of accelerometer-measured PA and sleep simultaneously. It is important to investigate not only the independent associations of time patterns of these lifestyle factors, but also how they interact to inform health outcomes.

Using data from the National Health and Nutrition Examination Survey (NHANES), this study aimed to identify diurnal patterns of PA and sleep in a US population, and to examine the independent and joint associations of these lifestyle patterns with all-cause mortality.

## Methods

### Study design and population

This study was conducted using individual-level data from NHANES, a program conducted by the National Centers for Health Statistics that recruited a representative sample of the civilian noninstitutionalized population in the US using a complex, multistage probability design. NHANES includes a series of cross-sectional surveys every two years since 1999. Data are collected through interviews and physical examinations, and mortality records have been linked from the date of survey participation to December 31, 2019. Further details of the protocol, study design, and data collection of NHANES are available elsewhere [23]. This study followed the Strengthening the Reporting of Observational Studies in Epidemiology (STROBE) guideline.

This study used data from the 2011–2012 and 2013–2014 cycles of NHANES because wearable accelerometer activity-rest monitoring was conducted during these rounds. There were 19,931 participants in the 2011–2014 cycles initially, and we included 9,342 non-pregnant participants aged ≥ 20 years who attended the accelerometer assessment. Further exclusion criteria included: (1) participants without at least three days valid accelerometer data (wear time ≥ 10 h for each day) (*n* = 475) [24]; (2) participants with no information on mortality (*n* = 18); and (3) participants with missing covariates (*n* = 2176). A total of 6,673 participants were enrolled in the final analyses (Supplementary Fig. 1).

### Accelerometery data management

The measurement of rest-activity status with accelerometers (ActiGraph model GT3X+) was implemented in NHANES 2011–2014. Participants were asked to wear devices on their non-dominant hand and told not to do anything with the device except to wear it. The devices measured acceleration (i.e., on the x-, y-, and z-axes) 80 times a second (80 Hz) and ambient light (lux) once a second (1 Hz). In this study, we used the summary datasets produced and released by NHANES, which included individual-level data summarized in monitor-independent movement summary units (MIMS-units) by minute, hour, and day [25, 26]. Although there are no established cut-off points for MIMS-units to distinguish the intensity of PA to date, MIMS units could be used to measure peak values which represent minute-level epochs with higher rates of movements across the period of monitoring [27]. An open-source algorithm was used to predict time periods of sleep wear, wake wear, or non-wear, and this algorithm has identified a category for each minute which was used to identify the diurnal patterns of sleep. This algorithm could reliably estimate movements and was sensitive in detecting subtle wrist movements during sedentary behaviours [25]. More details of the algorithm generation can be found in NHANES website [26].

The diurnal patterns of PA and sleep were identified using K-means clustering algorithm, which is an unsupervised machine learning algorithm that assigns participants into different clusters in which participants share similar characteristics. This algorithm has been used to identify diurnal patterns of accelerometer-measured lifestyle factors in previous studies [8, 10]. We computed the Euclidean distance as the similarity criterion [10]. For the diurnal pattern of PA, we used the proportions of total MIMS-units accumulated in each hour, with higher MIMS-units indicating higher intensity of PA. For sleep pattern, we calculated the proportions of accumulated sleep minutes in each hour as the independent variable. To ensure discriminability, interpretability and sufficient statistical power, the number of clusters was set at 3 (k-value = 3) for both PA and sleep (for 4 to 9 clusters, see illustrations in Supplementary Figs. 2 and 3).

### Mortality outcomes

The main outcome of this study was all-cause mortality, which was extracted from the National Death Index (NDI) dataset updated to December 31, 2019. In addition, we examined cause-specific mortality according to the following International Classification of Diseases 10th Revision (ICD-10): diabetes (E10-E14), CVD (I00-I09, I11, I13, I20-I51, I60-I69), cancer (C00-C97), and chronic lower respiratory diseases (J40-J47). Cardiometabolic mortality was defined as death due to diabetes or CVD.

### Covariates

Baseline covariates included age, sex (male/female), ethnicity (Mexican American/non-Hispanic White/non-Hispanic Black/other Hispanic/other), educational level (less than high school/high school or equivalent/college or above), marital status (married or living with partner/widowed/divorced or separated/never married), annual family income (0–19,999/20,000–44,999/45,000–74,999/75,000–99,999/100,000 and over), body mass index (BMI, underweight/normal weight/overweight/obese), alcohol intake (yes/no), current smoking status (yes/no), alternative healthy eating index (AHEI [28]), and history of diabetes, CVD, cancer, hypertension, and hyperlipidemia. Socioeconomic status (SES) was assessed using the summed score (0, 1, or 2) of educational level (0: less than college; 1: college or above) and annual family income (0: <45,000; 1: 45,000 and over), with a higher score indicating higher SES. We dichotomised the SES score into low or middle (0–1) versus high SES (2). Further covariates included total volume of PA, calculated as the sum of accelerometer measured MIMS-units per day, and total sleep duration, calculated as the sum hour of predicted sleep periods per day. We also derived self-reported moderate-to-vigorous physical activity (MVPA) and sedentary behaviour based on the Global Physical Activity Questionnaire [29]. Total amount of MVPA was calculated as the hours of metabolic equivalent (MET) per week following the NHANES guidance: PA (MET-h/week) = MET × weekly frequency × duration of each PA [3, 30]. Participants were divided into three groups of no PA (< 1 MET-h/week), low intensity PA (1–48 MET-h/week), and high intensity PA (> 48 MET-h/week) [31]. Time of participants’ sedentary behaviours per day were divided into quartiles: 0–4 h/day, 4–6 h/day, 6–9 h/day, and > 9 h/day. Self-reported sleep duration at night was categorized into < 6 h, 6–8 h, and > 8 h per day.

### Statistical analysis

All statistical analyses followed the complex sampling method with sample weight, stratification, and clustering in accordance with the NHANES analytic guidelines [32]. Characteristics of participant by PA and sleep diurnal patterns were presented as mean (standard deviation, SD) for continuous variables and numbers (percentage) for categorical variables, and were compared using analysis of variance adjusted for sampling weights and Rao-Scott χ^2^ tests.

We used the survey-weighted multinomial logistics regression models to assess factors associated with diurnal patterns of PA and sleep with all variables mutually adjusted in the same model. We used survey-weighted Cox proportional hazard models to examine the associations of diurnal patterns of PA, sleep, and their joint patterns with all-cause mortality. The proportional hazards assumption was tested by including product terms of follow-up time and PA and sleep patterns in the Cox model, and no significant deviation was found. Months from baseline to the date of death or end of follow-up (December 31, 2019), whichever occurred first, were calculated as the time scale. The fully adjusted model included age, sex, ethnicity, education level, marital status, family income, BMI, alcohol intake, smoking status, AHEI, disease history of diabetes, CVD, cancer, hypertension, hyperlipidemia, and accelerometer-measured PA volume and sleep duration per day. We adjusted for the diurnal sleep pattern in the analysis of PA, and diurnal pattern of PA in the analysis of sleep patterns. Stratified analyses were performed in subgroups of the following sociodemographic factors: sex (male and female), age (< 50 and ≥ 50 years), educational level (less than college and college or over), annual family income (< 45,000 and ≥ 45,000), and SES (low or middle and high).

A series of additional analyses were performed to test the robustness of the main results. First, we examined the associations of diurnal patterns of PA and sleep and their joint patterns with cause-specific mortality, including diabetes, CVD, cancer, cardiometabolic, chronic lower respiratory diseases, and other cause-specific mortality. Second, several sensitivity analyses were conducted after (1) including self-reported amount of MVPA, sedentary time, and sleep duration at night as covariates; (2) excluding participants with diabetes or CVD at baseline; (3) excluding participants with diabetes, CVD, cancer, hypertension, or hyperlipidemia at baseline; (4) excluding participants who reported no MVPA; (5) excluding participants with abnormal (< 1% or > 99%) accelerometer-measured PA volume or sleep duration; (6) excluding participants with less than two years follow-up; and (7) imputing missing covariates using multiple imputation for five times.

Odds ratios (ORs), hazard ratios (HRs) and 95% confidence intervals (95% CIs) were reported in this study. Significance tests were assessed at the 0.05 level using two-sided tests. All statistical analyses were performed using SAS (version 9.4, SAS Institute Inc., NC, USA) and R software (version 4.1.2).

## Results

### Population characteristics and diurnal patterns of physical activity and sleep

**Fig. 1:**
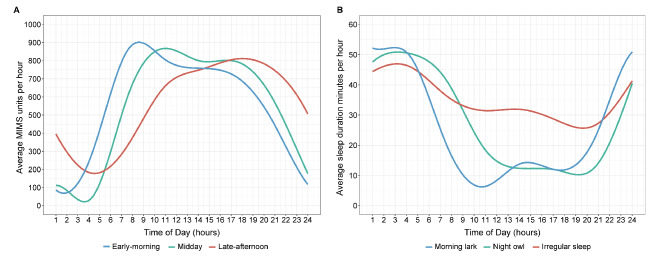
Diurnal patterns of physical activity (**A**) and sleep (**B**) **A**. Diurnal pattern of physical activity. Hourly monitor-independent movement summary (MIMS) units were calculated as the MIMS accumulated in each hour of the day. The average MIMS accumulated per hour across all subjects in each cluster was shown in different colours **B**. Diurnal pattern of sleep. Hourly sleep duration was calculated as the sleep duration accumulated in each hour of the day. The average sleep duration accumulated per hour across all subjects in each cluster was shown in different colours

Among the 6,673 participants included in this study, the mean age was 49.7 (SD = 17.4) years and 51.3% of participants were females. Figure 1 shows the three diurnal patterns of PA among the participants, including early-morning (*n* = 2,159, 32.4%), midday (*n* = 2,837, 42.5%), and late-afternoon (*n* = 1,677, 25.1%). The early-morning pattern had a remarkable spike of PA between 8:00 and 9:00, and the intensity of PA decreased gradually during the rest of the day. For the midday group, there was a long plateau period of moderate-to-high intensity PA ranging from 11:00 to 17:00, with the first peak intensity occurring at 10:00–12:00 and a second peak occurring at 16:00–17:00. The peak intensity of PA for participants with the late-afternoon pattern occurred at 17:00–19:00. The three diurnal patterns identified for sleep were: irregular sleep (*n* = 2,493, 37.4%), morning lark (*n* = 2,245, 33.6%), and night owl (*n* = 1,935, 29.0%). Participants with the morning lark pattern slept early at night and rose early in the morning, while those with the night owl pattern stayed up later at night and rose later in the morning. Participants with the irregular sleep pattern demonstrated varying times of sleep onset and awakening throughout the day, resulting in heightened nighttime sleep fragmentation and increased daytime sleepiness.

The characteristics of participants by the diurnal patterns of PA and sleep were shown in Table 1. Participants with the early-morning PA pattern were more likely to be older, males, non-Hispanic, less educated, married or living with partner, with higher family income, obese, non-smokers, and had higher rates of prior diabetes, CVD, hypertension, and hyperlipidemia, compared to those with the late-afternoon or midday pattern. Participants classified in the irregular sleep group were more likely to be males, non-Hispanic, divorced or separated, with low family income, obese, current smokers, and had higher rates of diabetes, CVD, and hypertension. They also had lower AHEI score, lower PA volume, and higher sleep duration per day, compared to those classified in the morning lark or night owl groups.

**Table 1:** Basic characteristics of participants by diurnal patterns of physical activity and sleep

Basic characteristics	Total (*n* = 6673)	Physical activity			*P* value	Sleep			*P* value
		Early-morning (*n* = 2159)	Late-afternoon (*n* = 1677)	Midday (*n* = 2837)		Irregular sleep (*n* = 2493)	Morning lark (*n* = 2245)	Night owl (*n* = 1935)	
**Age**,** mean (SD)**	49.7 (17.4)	54.2 (15.7)	41.6 (16.8)	51.0 (17.5)	< 0.001	49.3 (17.8)	53.6 (16.1)	45.5 (17.5)	< 0.001
**Sex**,** n (%)**					< 0.001				0.004
Male	3250 (48.7)	1102 (51.0)	852 (50.8)	1296 (45.7)		1309 (52.5)	1045 (46.5)	896 (46.3)	
Female	3423 (51.3)	1057 (49.0)	825 (49.2)	1541 (54.3)		1184 (47.5)	1200 (53.5)	1039 (53.7)	
**Ethnicity**,** n (%)**					< 0.001				< 0.001
Mexican American	722 (10.8)	267 (12.4)	133 (7.9)	322 (11.4)		205 (8.2)	294 (13.1)	223 (11.5)	
Non-Hispanic White	2948 (44.2)	997 (46.2)	640 (38.2)	1311 (46.2)		1077 (43.2)	1059 (47.2)	812 (42.0)	
Non-Hispanic Black	1536 (23.0)	509 (23.6)	476 (28.4)	551 (19.4)		692 (27.8)	421 (18.8)	423 (21.9)	
Other Hispanic	598 (9.0)	161 (7.5)	161 (9.6)	276 (9.7)		200 (8.0)	193 (8.6)	205 (10.6)	
Other	869 (13.0)	225 (10.4)	267 (15.9)	377 (13.3)		319 (12.8)	278 (12.4)	272 (14.1)	
**Educational level**,** n (%)**					0.019				0.342
Less than high school	1274 (19.1)	435 (20.1)	299 (17.8)	540 (19.0)		477 (19.1)	435 (19.4)	362 (18.7)	
High school or equivalent	1454 (21.8)	497 (23.0)	371 (22.1)	586 (20.7)		553 (22.2)	485 (21.6)	416 (21.5)	
College or above	3945 (59.1)	1227 (56.8)	1007 (60.0)	1711 (60.3)		1463 (58.7)	1325 (59.0)	1157 (59.8)	
**Marital status**,** n (%)**					< 0.001				< 0.001
Married or living with partner	3902 (58.5)	1366 (63.3)	765 (45.6)	1771 (62.4)		1260 (50.5)	1525 (67.9)	1117 (57.7)	
Widowed	494 (7.4)	200 (9.3)	73 (4.4)	221 (7.8)		199 (8.0)	199 (8.9)	96 (5.0)	
Divorced or separated	988 (14.8)	339 (15.7)	263 (15.7)	386 (13.6)		440 (17.6)	290 (12.9)	258 (13.3)	
Never married	1289 (19.3)	254 (11.8)	576 (34.3)	459 (16.2)		594 (23.8)	231 (10.3)	464 (24.0)	
**Annual family income**,** n (%)**					< 0.001				< 0.001
0–19,999	1610 (24.1)	410 (19.0)	544 (32.4)	656 (23.1)		726 (29.1)	399 (17.8)	485 (25.1)	
20,000–44,999	2072 (31.1)	644 (29.8)	545 (32.5)	883 (31.1)		816 (32.7)	646 (28.8)	610 (31.5)	
45,000–74,999	1220 (18.3)	443 (20.5)	250 (14.9)	527 (18.6)		407 (16.3)	466 (20.8)	347 (17.9)	
75,000–99,999	601 (9.0)	203 (9.4)	139 (8.3)	259 (9.1)		190 (7.6)	239 (10.6)	172 (8.9)	
100,000 and over	1170 (17.5)	459 (21.3)	199 (11.9)	512 (18.0)		354 (14.2)	495 (22.0)	321 (16.6)	
**Body mass index**,** n (%)**					< 0.001				< 0.001
Underweight	108 (1.6)	19 (0.9)	42 (2.5)	47 (1.7)		42 (1.7)	28 (1.2)	38 (2.0)	
Normal weight	1846 (27.7)	534 (24.7)	515 (30.7)	797 (28.1)		636 (25.5)	644 (28.7)	566 (29.3)	
Overweight	2139 (32.1)	720 (33.3)	478 (28.5)	941 (33.2)		771 (30.9)	781 (34.8)	587 (30.3)	
Obese	2580 (38.7)	886 (41.0)	642 (38.3)	1052 (37.1)		1044 (41.9)	792 (35.3)	744 (38.4)	
**Alcohol intake**,** n (%)**					0.110				0.878
No	1750 (26.2)	567 (26.3)	413 (24.6)	770 (27.1)		617 (24.7)	623 (27.8)	510 (26.4)	
Yes	4923 (73.8)	1592 (73.7)	1264 (75.4)	2067 (72.9)		1876 (75.3)	1622 (72.2)	1425 (73.6)	
**Smoking status**,** n (%)**					< 0.001				< 0.001
Never smokers	3767 (56.5)	1236 (57.2)	894 (53.3)	1637 (57.7)		1302 (52.2)	1351 (60.2)	1114 (57.6)	
Former smokers	1625 (24.4)	589 (27.3)	286 (17.1)	750 (26.4)		596 (23.9)	620 (27.6)	409 (21.1)	
Current smokers	1281 (19.2)	334 (15.5)	497 (29.6)	450 (15.9)		595 (23.9)	274 (12.2)	412 (21.3)	
**Diabetes**,** n (%)**					0.003				< 0.001
No	5775 (86.5)	1827 (84.6)	1488 (88.7)	2460 (86.7)		2077 (83.3)	1977 (88.1)	1721 (88.9)	
Yes	898 (13.5)	332 (15.4)	189 (11.3)	377 (13.3)		416 (16.7)	268 (11.9)	214 (11.1)	
**Cardiovascular disease**,** n (%)**					0.054				< 0.001
No	5975 (89.5)	1905 (88.2)	1536 (91.6)	2534 (89.3)		2170 (87.0)	2045 (91.1)	1760 (91.0)	
Yes	698 (10.5)	254 (11.8)	141 (8.4)	303 (10.7)		323 (13.0)	200 (8.9)	175 (9.0)	
**Cancer**,** n (%)**					< 0.001				0.010
No	6003 (90.0)	1915 (88.7)	1582 (94.3)	2506 (88.3)		2252 (90.3)	1984 (88.4)	1767 (91.3)	
Yes	670 (10.0)	244 (11.3)	95 (5.7)	331 (11.7)		241 (9.7)	261 (11.6)	168 (8.7)	
**Hypertension**,** n (%)**					< 0.001				< 0.001
No	4146 (62.1)	1201 (55.6)	1166 (69.5)	1779 (62.7)		1464 (58.7)	1354 (60.3)	1328 (68.6)	
Yes	2527 (37.9)	958 (44.4)	511 (30.5)	1058 (37.3)		1029 (41.3)	891 (39.7)	607 (31.4)	
**Hyperlipidemia**,** n (%)**					< 0.001				0.037
No	4209 (63.1)	1245 (57.7)	1225 (73.0)	1739 (61.3)		1563 (62.7)	1345 (59.9)	1301 (67.2)	
Yes	2464 (36.9)	914 (42.3)	452 (27.0)	1098 (38.7)		930 (37.3)	900 (40.1)	634 (32.8)	
**Alternative healthy eating index**,** n (%)**					< 0.001				< 0.001
Quartile 1	1668 (25.0)	456 (21.1)	567 (33.8)	645 (22.7)		730 (29.3)	422 (18.8)	516 (26.7)	
Quartile 2	1668 (25.0)	561 (26.0)	435 (25.9)	672 (23.7)		690 (27.7)	532 (23.7)	446 (23.0)	
Quartile 3	1668 (25.0)	573 (26.5)	344 (20.5)	751 (26.5)		590 (23.7)	591 (26.3)	487 (25.2)	
Quartile 4	1669 (25.0)	569 (26.4)	331 (19.7)	769 (27.1)		483 (19.4)	700 (31.2)	486 (25.1)	
**Total physical activity volume**,** n (%)**					0.063				< 0.001
Tertile 1	2224 (33.3)	690 (32.0)	544 (32.4)	990 (34.9)		1137 (45.6)	576 (25.7)	511 (26.4)	
Tertile 2	2224 (33.3)	718 (33.3)	536 (32.0)	970 (34.2)		763 (30.6)	811 (36.1)	650 (33.6)	
Tertile 3	2225 (33.3)	751 (34.8)	597 (35.6)	877 (30.9)		593 (23.8)	858 (38.2)	774 (40.0)	
**Total sleep duration per day**,** n (%)**					< 0.001				< 0.001
Tertile 1	2224 (33.3)	740 (34.3)	457 (27.3)	1027 (36.2)		148 (5.9)	1302 (58.0)	774 (40.0)	
Tertile 2	2224 (33.3)	716 (33.2)	560 (33.4)	948 (33.4)		522 (20.9)	795 (35.4)	907 (46.9)	
Tertile 3	2225 (33.3)	703 (32.6)	660 (39.4)	862 (30.4)		1823 (73.1)	148 (6.6)	254 (13.1)	

### Factors associated with diurnal patterns of physical activity and sleep

After mutually adjusted, several sociodemographic and lifestyle factors were associated with diurnal patterns of PA and sleep (Table 2). Compared to those with midday pattern, participants who were older; males; widowed, divorced or separated; or had higher family income were more likely to be physically active in early-morning. Compared to those with morning lark pattern, being younger; males; non-Hispanic Black; highly educated; single; with lower family income; obese; current smokers; and had lower AHEI score were associated with higher odds of experiencing irregular sleep. In addition, participants with diabetes (OR = 1.62, 95% CI = 1.23–2.13) or CVD (OR = 1.74, 95% CI = 1.28–2.38) were also at higher odds of experiencing irregular sleep.

**Table 2:** Factors associated with different diurnal patterns of physical activity and sleep

	Physical activity		Sleep	
	Early-morning	Late-afternoon	Irregular sleep	Night owl
**Age**	**1.01 (1.01–1.02)**	**0.97 (0.96–0.97)**	**0.98 (0.97–0.99)**	**0.97 (0.96–0.98)**
**Sex**				
Male	1.00 (Reference)	1.00 (Reference)	1.00 (Reference)	1.00 (Reference)
Female	**0.73 (0.60–0.89)**	**0.74 (0.64–0.85)**	**0.79 (0.66–0.95)**	1.03 (0.87–1.22)
**Ethnicity**				
Mexican American	1.02 (0.80–1.29)	0.79 (0.57–1.08)	0.77 (0.61–0.98)	0.93 (0.72–1.20)
Non-Hispanic White	1.00 (Reference)	1.00 (Reference)	1.00 (Reference)	1.00 (Reference)
Non-Hispanic Black	1.15 (0.95–1.41)	**1.46 (1.14–1.86)**	**1.29 (1.05–1.59)**	1.04 (0.85–1.26)
Other Hispanic	0.81 (0.62–1.06)	1.23 (0.94–1.62)	1.05 (0.76–1.45)	1.28 (0.88–1.87)
Other	0.86 (0.68–1.07)	**1.36 (1.03–1.79)**	1.28 (0.99–1.66)	1.12 (0.88–1.43)
**Educational level**				
Less than high school	1.00 (Reference)	1.00 (Reference)	1.00 (Reference)	1.00 (Reference)
High school or equivalent	1.26 (0.94–1.69)	1.09 (0.85–1.39)	1.07 (0.77–1.48)	0.95 (0.66–1.37)
College or above	0.87 (0.72–1.06)	1.15 (0.87–1.52)	**1.32 (1.01–1.72)**	1.07 (0.79–1.45)
**Marital status**				
Married or living with partner	1.00 (Reference)	1.00 (Reference)	1.00 (Reference)	1.00 (Reference)
Widowed	**1.27 (1.02–1.59)**	**1.46 (1.07-2.00)**	**1.48 (1.10–1.99)**	0.96 (0.70–1.30)
Divorced or separated	**1.42 (1.11–1.81)**	**1.61 (1.17–2.21)**	**1.50 (1.16–1.93)**	1.11 (0.85–1.44)
Never married	0.90 (0.72–1.13)	**1.66 (1.27–2.18)**	**2.30 (1.86–2.84)**	**1.81 (1.34–2.44)**
**Annual family income**				
0-19999	**0.47 (0.38–0.59)**	**1.40 (1.05–1.87)**	**2.00 (1.46–2.75)**	**1.68 (1.26–2.22)**
20,000–44,999	**0.70 (0.55–0.88)**	**1.36 (1.00-1.85)**	**1.57 (1.22–2.01)**	**1.30 (1.00-1.69)**
45,000–74,999	0.87 (0.69–1.11)	1.03 (0.77–1.37)	1.13 (0.82–1.54)	1.02 (0.76–1.38)
75,000–99,999	0.87 (0.65–1.16)	1.15 (0.82–1.62)	1.14 (0.86–1.52)	0.93 (0.68–1.26)
100,000 and over	1.00 (Reference)	1.00 (Reference)	1.00 (Reference)	1.00 (Reference)
**Body mass index**				
Underweight	0.50 (0.22–1.12)	1.11 (0.62–2.01)	1.44 (0.85–2.44)	1.37 (0.77–2.45)
Normal weight	1.00 (Reference)	1.00 (Reference)	1.00 (Reference)	1.00 (Reference)
Overweight	1.03 (0.85–1.24)	0.92 (0.72–1.18)	1.13 (0.92–1.39)	0.99 (0.80–1.22)
Obese	1.17 (0.91–1.49)	1.11 (0.84–1.45)	**1.35 (1.05–1.75)**	1.14 (0.90–1.44)
**Alcohol intake**				
No	1.00 (Reference)	1.00 (Reference)	1.00 (Reference)	1.00 (Reference)
Yes	1.03 (0.86–1.22)	0.92 (0.76–1.12)	1.00 (0.80–1.25)	0.95 (0.79–1.13)
**Smoking status**				
Never smokers	1.00 (Reference)	1.00 (Reference)	1.00 (Reference)	1.00 (Reference)
Former smokers	**0.79 (0.67–0.94)**	0.98 (0.76–1.26)	1.01 (0.85–1.20)	0.92 (0.76–1.11)
Current smokers	0.99 (0.76–1.28)	**1.66 (1.28–2.16)**	**1.89 (1.48–2.42)**	**1.62 (1.31–1.99)**
**Alternative healthy eating index**				
Quartile 1	1.00 (Reference)	1.00 (Reference)	1.00 (Reference)	1.00 (Reference)
Quartile 2	1.04 (0.89–1.22)	0.99 (0.80–1.22)	1.01 (0.85–1.21)	0.95 (0.77–1.18)
Quartile 3	1.00 (0.86–1.17)	**0.76 (0.60–0.97)**	0.84 (0.67–1.07)	0.99 (0.81–1.21)
Quartile 4	0.98 (0.78–1.22)	0.86 (0.65–1.13)	**0.65 (0.50–0.84)**	0.92 (0.70–1.21)
**Diabetes**				
No	1.00 (Reference)	1.00 (Reference)	1.00 (Reference)	1.00 (Reference)
Yes	1.21 (0.93–1.57)	1.38 (0.96–1.98)	**1.62 (1.23–2.13)**	1.25 (0.96–1.63)
**Cardiovascular disease**				
No	1.00 (Reference)	1.00 (Reference)	1.00 (Reference)	1.00 (Reference)
Yes	0.76 (0.55–1.06)	1.19 (0.86–1.63)	**1.74 (1.28–2.38)**	**1.73 (1.30–2.31)**
**Cancer**				
No	1.00 (Reference)	1.00 (Reference)	1.00 (Reference)	1.00 (Reference)
Yes	0.81 (0.62–1.05)	0.86 (0.64–1.17)	0.98 (0.75–1.29)	1.19 (0.87–1.63)
**Hypertension**				
No	1.00 (Reference)	1.00 (Reference)	1.00 (Reference)	1.00 (Reference)
Yes	1.09 (0.94–1.26)	1.03 (0.84–1.26)	1.14 (0.88–1.48)	0.97 (0.80–1.18)
**Hyperlipidemia**				
No	1.00 (Reference)	1.00 (Reference)	1.00 (Reference)	1.00 (Reference)
Yes	0.94 (0.82–1.07)	0.86 (0.68–1.09)	1.14 (0.95–1.37)	**1.26 (1.04–1.54)**

All factors were mutually adjusted in the same model

Midday and morning lark groups were considered as the reference groups for the analyses of physical activity and sleep, respectively

### Diurnal patterns of physical activity, sleep, and their joint associations with all-cause mortality

During the median follow-up of 6.8 years (interquartile range 5.8–7.8), 589 participants died. The independent and joint associations of diurnal patterns of PA and sleep with all-cause mortality are presented in Table 3. After adjusting for sociodemographic and lifestyle factors (Model 2), the risk of all-cause mortality was 33% higher for participants with the early-morning PA pattern (HR = 1.33, 95% CI = 1.11–1.60) and 44% higher for participants with the irregular sleep pattern (HR = 1.44, 95% CI = 1.09–1.90), compared to those with a midday PA and morning lark pattern, respectively. These associations were robust to additional adjustment for the accelerometer-measured PA volume and sleep duration per day (early-morning: HR = 1.36, 95% CI = 1.13–1.64; irregular sleep: HR = 1.42, 95% CI = 1.01–1.99). For the joint diurnal patterns of PA and sleep, participants with an early-morning and irregular sleep pattern had higher risk of all-cause mortality compared to those with a midday and morning lark pattern (HR = 1.92, 95% CI = 1.33–2.78).

**Table 3:** Independent and joint associations of diurnal patterns of physical activity and sleep with all-cause mortality

	Case/*N*	Model 1	Model 2	Model 3
**Physical activity**				
Early-morning	242/2159	**1.30 (1.06–1.59)**	**1.33 (1.11–1.60)**	**1.36 (1.13–1.64)**
Late-afternoon	112/1677	1.44 (0.97–2.15)	1.19 (0.84–1.69)	1.21 (0.85–1.71)
Midday	235/2837	1.00 (Reference)	1.00 (Reference)	1.00 (Reference)
**Sleep**				
Irregular sleep	292/2493	**1.78 (1.36–2.33)**	**1.44 (1.09–1.90)**	**1.42 (1.01–1.99)**
Morning lark	193/2245	1.00 (Reference)	1.00 (Reference)	1.00 (Reference)
Night owl	104/1935	1.06 (0.68–1.65)	1.01 (0.64–1.58)	1.02 (0.65–1.60)
**Joint pattern of physical activity and sleep**				
Early-morning & Morning lark	109/1146	1.04 (0.77–1.40)	1.09 (0.80–1.47)	1.11 (0.82–1.51)
Early-morning & Night owl	13/246	1.11 (0.46–2.71)	1.17 (0.47–2.92)	1.24 (0.49–3.10)
Early-morning & Irregular sleep	120/767	**2.32 (1.65–3.25)**	**1.91 (1.37–2.67)**	**1.92 (1.33–2.78)**
Late-afternoon & Morning lark	3/59	2.75 (0.93–8.16)	2.31 (0.83–6.41)	2.50 (0.91–6.86)
Late-afternoon & Night owl	31/750	1.25 (0.75–2.09)	0.97 (0.56–1.68)	1.00 (0.58–1.74)
Late-afternoon & Irregular sleep	78/868	**2.29 (1.43–3.65)**	1.57 (0.98–2.53)	1.56 (0.94–2.58)
Midday & Morning lark	81/1040	1.00 (Reference)	1.00 (Reference)	1.00 (Reference)
Midday & Night owl	60/939	1.02 (0.63–1.66)	0.98 (0.58–1.63)	0.99 (0.59–1.65)
Midday & Irregular sleep	94/858	1.38 (0.94–2.02)	1.14 (0.75–1.73)	1.13 (0.74–1.71)

Model 1: adjusted for age and sex

Model 2: additionally adjusted for ethnicity, educational level, marital status, family income, body mass index, alcohol intake, smoking status, alternative healthy eating index, diabetes, cardiovascular disease, cancer, hypertension, and hyperlipidaemia based on model 1

Model 3: additionally adjusted for accelerometer-measured physical activity volume and sleep duration per day based on model 2

### Differences by sociodemographic factors in the associations with all-cause mortality

**Fig. 2:**
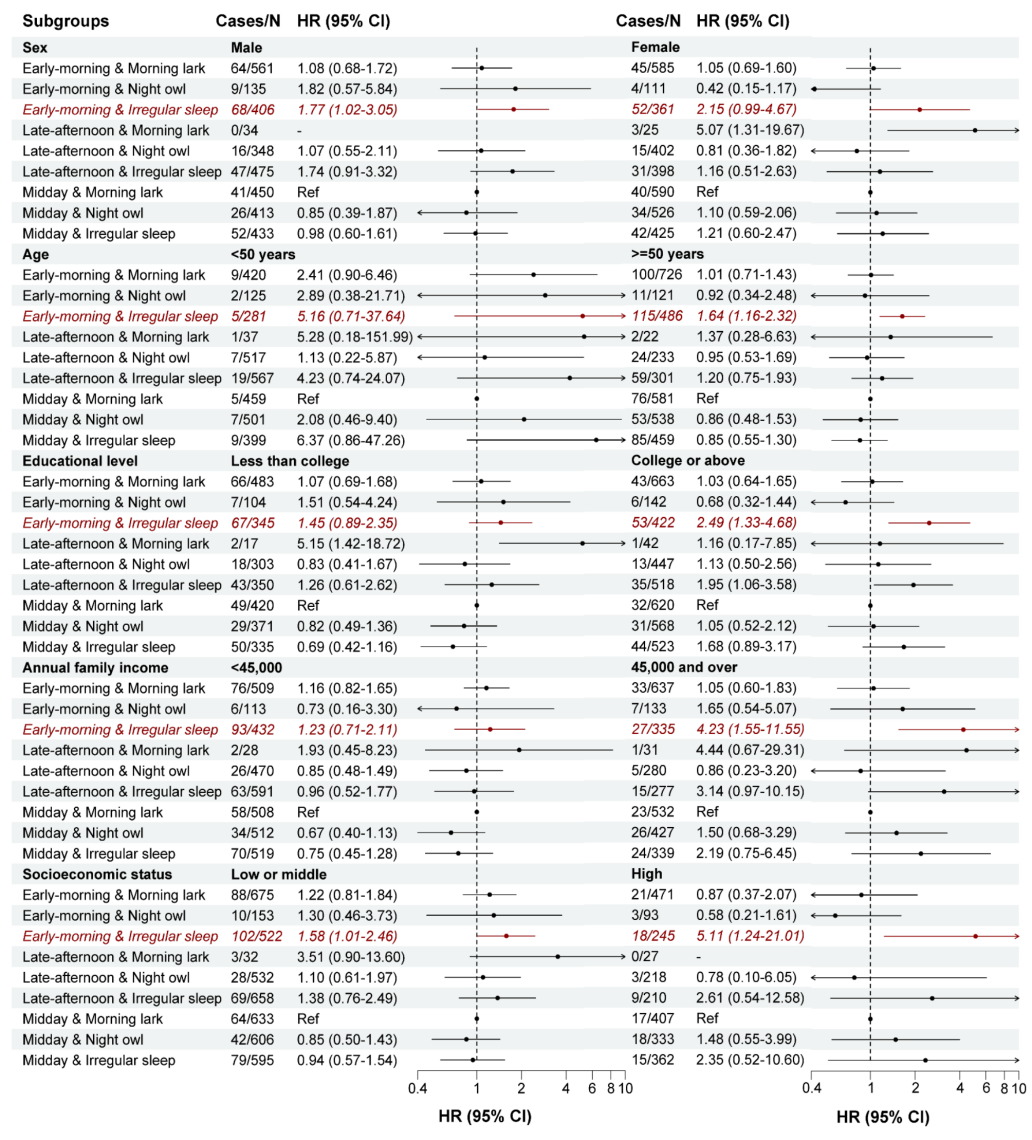
Subgroup analysis of the joint associations of diurnal patterns of physical activity and sleep with all-cause mortality

In all stratified analyses by sex, age, educational level, annual family income, and SES, the HRs for early-morning PA and irregular sleep, independently and jointly, exceeded one (range 1.23 to 5.16) (Fig. 2, Supplementary Figs. 4 and 5). The association between early-morning PA pattern and all-cause mortality was statistically significant among participants who were males, older, non-Hispanic white, less educated, with lower family income, and low or middle SES (Supplementary Fig. 4). The association between irregular sleep and all-cause mortality was statistically significant among those who were non-Hispanic white, higher educated, with higher family income and higher SES (Supplementary Fig. 5). For the joint pattern of PA and sleep, the association of the combination of early-morning PA and irregular sleep with all-cause mortality reached statistical significance among participants who were males, older, with higher education, or with higher family income and higher SES (Fig. 2).

### Cause-specific mortality and sensitivity analyses

The associations of early-morning PA pattern, irregular sleep pattern and their joint patterns with mortality caused by diabetes, CVD, cancer, cardiometabolic diseases, chronic lower respiratory and other causes were consistent with those observed for all-cause mortality (Supplementary Tables 1–2). The sensitivity analyses also showed similar results to our main findings after exclusion of specific participant groups (Supplementary Tables 3–8). Besides, when we included participants with missing covariates in the final analysis, diurnal patterns of PA and sleep identified were consistent with the main analysis (Supplementary Fig. 6). After imputing the missing data, the associations of diurnal patterns of PA and sleep and their combinations with all-cause mortality were similar to the main results (Supplementary Table 9).

## Discussion

In this prospective study of a US population, we identified three diurnal patterns for both of accelerometer-measured PA and sleep: peak PA in early-morning, midday, and late-afternoon and irregular sleep, morning lark, and night owl sleep patterns. Participants who were older; males; widowed, divorced or separated or had higher family income were more likely to be physically active in early-morning, while those who were younger; males; non-Hispanic Black; single; with higher educational level; lower family income; obese; current smokers; low scorers in AHEI; or had diabetes or CVD were more likely to experience irregular sleep. The early-morning PA pattern, irregular sleep pattern, and their joint patterns were all associated with higher risk of all-cause mortality, independent of the volume of PA, sleep duration, and sociodemographic and health-related covariates. The lowest mortality risk was observed for midday physical activity combined with the morning lark sleep pattern, midday physical activity combined with the night owl pattern, late-afternoon physical activity combined with the night owl pattern, and early-morning physical activity combined with the morning lark pattern. Moreover, while sociodemographic differences existed among the associations between early-morning PA, irregular sleep and all-cause mortality, the effect estimates favoured increased risk for these specific diurnal patterns in all subgroups.

### Comparison with other studies

The three diurnal patterns of PA we identified in a US population (2011–2014) are in agreement with those reported in a previous study based on MVPA in NHANES (2003–2006) [10]. To minimise potential bias arising from the influence of intensity levels of PA and sedentary behaviours [33], we conducted two sensitivity analyses by including self-reported amount of MVPA and sedentary time as covariates and by excluding participants who reported no MVPA. The results remained similar, suggesting that different intensities of PA and sedentary time are not driving our findings. Our findings were also robust to exclusions of participants with abnormal (< 1% or > 99%) accelerometer-measured physical activity volume or sleep duration, those with less than 2 years of follow-up and individuals with diabetes, CVD, cancer, hypertension or hyperlipidemia at baseline. This suggests that outliers and reverse causation due to pre-existing disease are unlikely explanations for our findings.

Our results on early-morning PA and mortality from CVD are in line with previous population-based observational findings [8, 9, 33, 34]. A cross-sectional study, for example, reported that being most active in the morning was associated with higher risk of CVD [33]. A prospective cohort study based on a UK population found a 1.56-fold increased risk of CVD mortality for participants in the early-morning PA group (peak intensity occurred at 7:00–9:00) compared to those in the midday group (peak intensity occurred at 10:00–13:00) [8]. In a large-scale prospective study based on 92,139 UK Biobank participants, the risk of all-cause and CVD mortality was lower in the midday-afternoon group than those in the morning group [9]. Consistent results were also reported in several randomized controlled trials [7, 35].

The unfavourable health effects of early-morning PA could be partially explained by the rapid increase of blood pressure accompanied with PA and the increased blood pressure reactivity to physical exertion in the early morning [36]. However, some conflicting results exist [11, 13]. A prospective cohort study based on 86,657 populations from UK Biobank suggests that morning physical activity was associated with lower risks of CVD [11]. Similarly, another study of UK Biobank populations (*n* = 93,095) found a protective effect of physical activity in the morning on diabetes risk [13]. These inconsistent results might partly result from (1) different study outcomes; (2) varied study populations; (3) different measurement methods of physical activity (ActiGraph model GT3X + in NHANES and Axivity AX3 in UK Biobank) and physical activity patterns. It is important to extend the concept of chronoactivity in further studies to verify the results.

This study also found three patterns of sleep using accelerometer-measured data. Unlike previous studies reporting that evening chronotype (i.e., get up and go to bed late) was associated with higher risk of morbidity and mortality compared with morning chronotype (i.e., get up and go to bed early) [37–39], our results showed no statistically significant association between the night owl pattern and mortality. The inconsistent results might partly attribute to the usage of self-reported question-defining sleep chronotypes in previous studies and different study populations. Our study showed that participants with the irregular sleep pattern were at higher risk of all-cause mortality, suggesting the sleep regularity might be a more important predictor of health than a consistently early or late onset of sleep. In line with our results, previous studies using self-reported sleep data have linked irregular sleep patterns with adverse health outcomes such as metabolic syndrome [40] and all-cause mortality [41], and such associations were independent of sleep durations. Other studies using accelerometer-measured data also provided similar evidence that sleep irregularity is an important predictor of reduced quality of life [18] and higher risk of mortality [17].

Participants in the irregular sleep pattern are characterised by increased nighttime sleep fragmentation and daytime sleepiness. Consequently, they are more likely to be exposed to irregular environmental stimulations, potentially resulting in the disruption of circadian rhythms and downstream adverse health outcomes [42, 43]. This interpretation is supported by animal studies showing that circadian disruption induced by irregular sleep is a causal risk factor of premature mortality [44].

Moreover, our results showed that participants with prior CVD and diabetes were at particularly high risk of experiencing irregular sleep, which could also contribute to the higher risk of mortality for participants in this group [45]. However, due to the difficulty of distinguishing the onset time of diseases and sleep irregularity, the temporal relationship could not be reliably inferred in the present study. This issue warrants future studies to validate such mechanisms.

We conducted joint analyses of the diurnal patterns of PA and sleep to address potential complex interactions between two behaviours. Our results supported a significant joint association of the diurnal patterns of PA and sleep with all-cause mortality which was stronger than the independent associations. Previous studies have suggested that higher intensity PA and optimal sleep duration in combination were associated with delayed cognitive decline, better mental health, improved cardiometabolic health, and reduced risk of all-cause, CVD, and cancer mortality across different age groups [21, 22, 46–48]. Our study extends previous evidence by capturing 9 different combinations of diurnal patterns and by demonstrating that the independent effects of diurnal PA and sleep patterns would be exaggerated among participants with both early-morning PA and irregular sleep. To the best of our knowledge, this is the first study examining the joint association of their diurnal patterns with mortality risk. Our findings, taken together with existing evidence, suggest that future lifestyle-based intervention strategies should consider the diurnal patterns of both PA and sleep, in addition to their volume, frequency and duration, to maximise the health benefits.

We observed several sociodemographic differences in the diurnal patterns of PA and sleep and their associations with mortality. The crucial role of sociodemographic factors in the associations of PA and sleep with health outcomes have been reported in previous studies [33, 49–56]. For example, it was suggested that the male morning PA groups had higher risk of developing CVD than the corresponding female groups, which could be partly explained by the difference of VO_2max_ in response to the endurance training between men and women [33, 57]. For the role of age on circadian rhythm, studies have reported that older adults were more likely to be active in the morning and to have earlier circadian phase [50, 58]. This finding is consistent with our results and the suggestion that these people are more active during the night and less active during the day, resulting increased sleep fragmentation and daytime sleepiness with age [59, 60].

Interestingly, we found that the role of SES in the association between PA with mortality differed from that in the association between sleep with mortality. Thus, the association between early-morning PA pattern and mortality was stronger and that between irregular sleep and mortality weaker among participants with low or middle SES compared to those with high SES. According to our results on factors associated with PA and sleep patterns, participants with lower income were less likely to be physically active in the morning, but were more likely to experience irregular sleep. Several factors could explain these differences, including work stress, work schedules, and work-life imbalance. As a novel observation, it remains unknown why this preference difference existed. These sociodemographic variations imply that the policies targeting diurnal patterns of PA and sleep might have different impact in population sub-groups.

### Strength and limitations

The main strengths of this study include its prospective study design, the accelerometer-measured PA and sleep, the usage of a machine learning algorithm to classify diurnal patterns, and the opportunity to adjust the effect estimates for multiple covariates. In addition, the sensitivity analyses confirmed the robustness of our results. Several limitations should also be acknowledged. First, there is currently a lack of established cut-off points for MIMS-units to determine levels of PA intensity. Therefore, we could not identify MVPA. Also, the recreational PA and occupational PA could not be differentiated. An algorithm to identify different intensity levels of PA is warranted for future analysis which would allow researchers to consider more detailed characteristics of PA. Second, although accelerometer-based measurement is widely used to determine sleep status [61, 62], the measure might overestimate sleep duration [63]. Moreover, the correlation of accelerometer-measured and self-reported sleep duration was low in NHANES [64]. To address this issue, we included self-reported amount of MVPA and sleep duration as covariates in the sensitivity analysis; the results did not materially change. Nonetheless, there remains a need to test the accuracy of the accelerometer-derived sleep and wake categories in NHANES. Third, as our study is based on observational data, we could not eliminate residual confounding even after adjusting for known covariates. Fourth, the sample size of this study is relatively small, limiting the generalizability of our results. The sample sizes in the subgroup analyses of the joint association of diurnal patterns of PA and sleep were particularly small, resulting in a large 95% CI for some subcategories. Fifth, the observational study design cannot demonstrate the causality relationship. Considering these limitations, further studies are warranted to validate the observed associations with larger sample sizes and, if feasible, using randomized controlled trial design.

### Implications

This study has critical public health implications. PA and sleep have already been identified as two important targets for the prevention and intervention of major health problems by the World Health Organization guidelines [1]. Beyond previous suggestions that focused on PA volume and intensity as well as sleep duration, our study highlights the importance of the timing and diurnal patterns of lifestyle behaviours in disease prevention and health management. As the evidence on the effects of timing of these behaviours on different health outcomes remain relatively limited, future research is needed to validate the present evidence for health policies. In these interventional studies, it is particularly important to examine the effectiveness of simultaneously targeting the timing of PA and sleep to maximize the health benefits [21, 22]. Furthermore, considering the sociodemographic differences identified on the diurnal patterns of PA and sleep and their association with all-cause mortality, lifestyle-based health promotion strategies may need to be tailored for population sub-groups.

## Conclusions

In this population-based prospective cohort study, we found that early-morning PA pattern and irregular sleep pattern were associated with higher risk of all-cause mortality. These independent associations were strengthened in individuals with a combined diurnal pattern of early-morning PA and irregular sleep. Our study suggests that diurnal patterns of physical activity and sleep, in addition to their duration and frequency, may play an important role in lifestyle-based health promotion strategies.

## Electronic supplementary material

Below is the link to the electronic supplementary material.

Supplementary Material 1

Supplementary Material 2

## Data Availability

The original data for the study are available on the NHANES website: https://wwwn.cdc.gov/nchs/nhanes/Default.aspx.

## Abbreviations

AHEI: alternative healthy eating index
CI: confidence interval
CVD: cardiovascular diseases
HR: hazard ratio
ICD-10: International Classification of Diseases 10th Revision
MIMS: monitor-independent movement summary
MVPA: moderate-to-vigorous physical activity
NDI: National Death Index
NHANES: National Health and Nutrition Examination Survey
OR: odds ratio
PA: physical activity
SD: standard deviation
SES: socioeconomic status
T2D: type 2 diabetes
