# Correction: Diurnal patterns of accelerometer-measured physical activity and sleep and risk of all-cause mortality: a follow-up of the National Health and Nutrition Examination Surveys (NHANES)

**DOI:** 10.1186/s12966-024-01680-w

**Published:** 2024-11-11

**Authors:** Yue Zhang, Mika Kivimäki, Rodrigo M. Carrillo-Larco, Yangyang Cheng, Yaguan Zhou, Hui Wang, Changzheng Yuan, Xiaolin Xu

**Affiliations:** 1 School of Public Health, The Second Affiliated Hospital, Zhejiang University School of Medicine, Hangzhou, Zhejiang 310058 China; 2 The Key Laboratory of Intelligent Preventive Medicine of Zhejiang Province, Hangzhou, China; 3 UCL Brain Sciences, University College London, London, UK; 4 Emory Global Diabetes Research Center, Emory University, Atlanta, GA USA; 5 Hubert Department of Global Health, Rollins School of Public Health, Emory University, Atlanta, GA USA; 6 Department of Nutrition, Harvard T.H. Chan School of Public Health, Boston, MA USA; 7 School of Public Health, Faculty of Medicine, The University of Queensland, Brisbane, Australia

**Correction: Zhang et al. International Journal of Behavioral Nutrition and Physical Activity 21:120 (2024)**

10.1186/s12966-024-01673-9

Following the publication of the original article, the authors reported errors in the legend of Fig. 1.


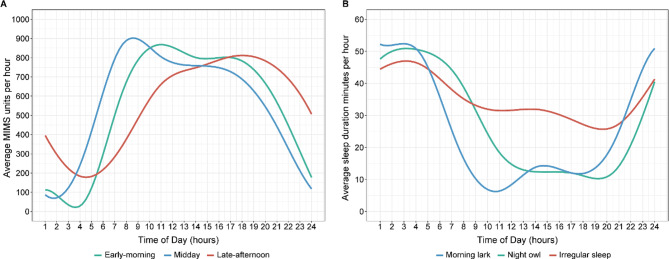


The legends of early-morning physical activity and midday physical activity have been wrongly swapped in Fig. 1 (A) – the blue and green line.

The correct legend is as follows:


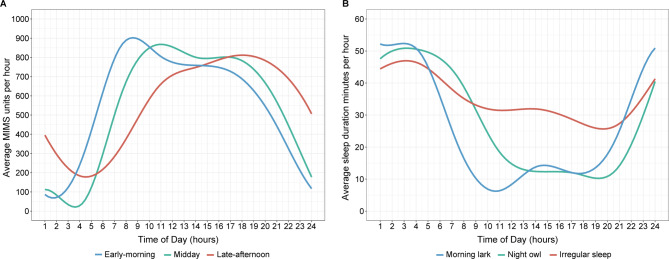


The original article has been updated.

