# Supplementary material for: Diurnal patterns of accelerometer-measured physical activity and sleep and risk of all-cause mortality: a follow-up of the National Health and Nutrition Examination Surveys (NHANES)
Source: Int J Behav Nutr Phys Act. 2024 Oct 18;21:120. doi: 10.1186/s12966-024-01673-9 (PMC11490014; doi:10.1186/s12966-024-01673-9)
Supplement: Supplementary file 2 — Supplementary Material 2 [file 12966_2024_1673_MOESM2_ESM.docx]

Content

[Supplementary Tables 2](#_Toc171972426)

[**Supplementary Table 1.** Associations of diurnal patterns of physical activity and sleep with specific-cause mortality. 2](#_Toc171972427)

[**Supplementary Table 2.** Joint associations of diurnal patterns of physical activity and sleep with specific-cause mortality. 3](#_Toc171972428)

[**Supplementary Table 3.** Sensitivity analysis of the associations of diurnal patterns of physical activity and sleep with all-cause mortality by including self-reported amount of moderate and vigorous physical activity, sedentary time, and sleep duration at night as covariates. 4](#_Toc171972429)

[**Supplementary Table 4.** Sensitivity analysis of the associations of diurnal patterns of physical activity and sleep with all-cause mortality by excluding participants with diabetes or CVD at baseline (N=5329). 5](#_Toc171972430)

[**Supplementary Table 5.** Sensitivity analysis of the associations of diurnal patterns of physical activity and sleep with all-cause mortality by excluding participants with diabetes, CVD, cancer, hypertension or hyperlipidemia at baseline (N=2819). 6](#_Toc171972431)

[**Supplementary Table 6.** Sensitivity analysis of the associations of diurnal patterns of physical activity and sleep with all-cause mortality by excluding participants who reported no moderate-to-vigorous physical activity (n=6657). 7](#_Toc171972432)

[**Supplementary Table 7.** Sensitivity analysis of the associations of diurnal patterns of physical activity and sleep with all-cause mortality by excluding participants with abnormal (<1% or >99%) accelerometer-measured physical activity volume or sleep duration (n=6425). 8](#_Toc171972433)

[**Supplementary Table 8.** Sensitivity analysis of the associations of diurnal patterns of physical activity and sleep with all-cause mortality by excluding participants with less than 2 years follow-up (n=6,566). 9](#_Toc171972434)

[**Supplementary Table 9.** Sensitivity analysis of the associations of diurnal patterns of physical activity and sleep with all-cause mortality by imputing missing covariates (n=8,849). 10](#_Toc171972435)

[Supplementary Figures 11](#_Toc171972436)

[**Supplementary Figure 1.** Flow chart of the selection of study population. 11](#_Toc171972437)

[**Supplementary Figure 2.** Diurnal patterns of physical activity by machine learning-based clustering using a higher number of clusters (k=4~9). 12](#_Toc171972438)

[**Supplementary Figure 3.** Diurnal patterns of sleep by machine learning-based clustering using a higher number of clusters (k=4~9). 13](#_Toc171972439)

[**Supplementary Figure 4.** Subgroup analysis for the associations of diurnal patterns of physical activity with all-cause mortality. 14](#_Toc171972440)

[**Supplementary Figure 5.** Subgroup analysis for the associations of diurnal patterns of sleep with all-cause mortality 15](#_Toc171972441)

[**Supplementary Figure 6.** Sensitivity analysis of diurnal patterns of physical activity (A) and sleep (B) by including participants with missing covariates. 16](#_Toc171972442)

# Supplementary Tables

## **Supplementary Table 1.** Associations of diurnal patterns of physical activity and sleep with specific-cause mortality.

|  | **Diabetes** | | **CVD** | | **Cancer** | | **Cardiometabolic** | | **Chronic lower respiratory diseases** | | **Other** | |
| --- | --- | --- | --- | --- | --- | --- | --- | --- | --- | --- | --- | --- |
|  | **Case/N** | **HR (95% CI)** | **Case/N** | **HR (95% CI)** | **Case/N** | **HR (95% CI)** | **Case/N** | **HR (95% CI)** | **Case/N** | **HR (95% CI)** | **Case/N** | **HR (95% CI)** |
| **Physical activity** | | | | | | | | | | | | |
| Early-morning | 13/2159 | 1.82 (0.52-6.35) | 89/2159 | 1.25 (0.83-1.90) | 55/2159 | 1.41 (0.83-2.41) | 102/2159 | 1.36 (0.95-1.95) | 14/2159 | 1.55 (0.58-4.18) | 46/2159 | 1.50 (0.80-2.80) |
| Late-afternoon | 7/1677 | 0.68 (0.21-2.21) | 28/1677 | 0.89 (0.36-2.22) | 27/1677 | 1.43 (0.59-3.47) | 35/1677 | 0.94 (0.45-1.97) | 5/1677 | 1.10 (0.20-6.17) | 32/1677 | 1.64 (0.84-3.19) |
| Midday | 14/2837 | Ref | 71/2837 | Ref | 64/2837 | Ref | 85/2837 | Ref | 15/2837 | Ref | 50/2837 | Ref |
| **Sleep** |  |  |  |  |  |  |  |  |  |  |  |  |
| Irregular sleep | 21/2493 | 11.0 (1.97-61.9) | 99/2493 | 1.12 (0.58-2.17) | 65/2493 | 1.45 (0.79-2.68) | 120/2493 | 1.46 (0.82-2.58) | 14/2493 | 0.90 (0.26-3.07) | 61/2493 | 1.44 (0.54-3.85) |
| Morning lark | 8/2245 | Ref | 67/2245 | Ref | 48/2245 | Ref | 75/2245 | Ref | 14/2245 | Ref | 40/2245 | Ref |
| Night owl | 5/1935 | 1.91 (0.35-10.5) | 22/1935 | 0.68 (0.27-1.69) | 33/1935 | 1.26 (0.66-2.43) | 27/1935 | 0.73 (0.32-1.71) | 6/1935 | 1.51 (0.48-4.78) | 27/1935 | 0.92 (0.34-2.51) |

All models were adjusted for age, sex, race, educational level, marital status, family income, body mass index, alcohol intake, smoking status, alternative healthy eating index, total energy intake, diabetes, cardiovascular disease, cancer, hypertension, hyperlipidaemia, accelerometer-measured physical activity volume and sleep duration.

## **Supplementary Table 2.** Joint associations of diurnal patterns of physical activity and sleep with specific-cause mortality.

|  | **Diabetes** | | **CVD** | | **Cancer** | | **Cardiometabolic** | | **Chronic lower respiratory diseases** | | **Other** | |
| --- | --- | --- | --- | --- | --- | --- | --- | --- | --- | --- | --- | --- |
|  | **Case/N** | **HR (95% CI)** | **Case/N** | **HR (95% CI)** | **Case/N** | **HR (95% CI)** | **Case/N** | **HR (95% CI)** | **Case/N** | **HR (95% CI)** | **Case/N** | **HR (95% CI)** |
| Early-morning & Morning lark | 6/1146 | 2.71 (0.26-28.4) | 39/1146 | 0.92 (0.43-1.96) | 29/1146 | 1.66 (0.91-3.03) | 45/1146 | 1.01 (0.52-1.96) | 6/1146 | 0.74 (0.20-2.76) | 21/1146 | 1.01 (0.56-1.82) |
| Early-morning & Night owl | 0/246 | - | 3/246 | 0.40 (0.11-1.44) | 1/246 | 1.41 (0.16-12.3) | 3/246 | 0.40 (0.11-1.36) | 0/246 | - | 6/246 | 2.23 (0.38-13.0) |
| Early-morning & Irregular sleep | 7/767 | **25.4 (2.75-235)** | 47/767 | 1.34 (0.56-3.19) | 25/767 | 2.38 (0.93-6.07) | 54/767 | 1.85 (0.92-3.71) | 8/767 | 2.00 (0.59-6.75) | 19/767 | 1.69 (0.47-6.05) |
| Late-afternoon & Morning lark | 0/59 | - | 0/59 | - | 1/59 | 4.50 (0.71-28.5) | 0/59 | - | 1/59 | 30.7 (8.42-112) | 0/59 | - |
| Late-afternoon & Night owl | 2/750 | 1.59 (0.24-10.4) | 4/750 | 0.27 (0.07-1.05) | 12/750 | 2.57 (0.83-7.95) | 6/750 | 0.32 (0.09-1.12) | 1/750 | 0.88 (0.09-8.26) | 9/750 | 0.99 (0.30-3.27) |
| Late-afternoon & Irregular sleep | 5/868 | **9.65 (1.20-77.4)** | 24/868 | 1.00 (0.44-2.29) | 14/868 | 1.63 (0.48-5.54) | 29/868 | 1.34 (0.61-2.92) | 3/868 | 0.64 (0.08-4.78) | 23/868 | 2.08 (0.79-5.47) |
| Midday & Morning lark | 2/1040 | Ref | 28/1040 | Ref | 18/1040 | Ref | 30/1040 | Ref | 7/1040 | Ref | 19/1040 | Ref |
| Midday & Night owl | 3/939 | 3.48 (0.61-19.8) | 15/939 | 0.74 (0.30-1.88) | 20/939 | 1.36 (0.57-3.26) | 18/939 | 0.82 (0.33-2.04) | 5/939 | 1.95 (0.60-6.37) | 12/939 | 0.58 (0.24-1.39) |
| Midday & Irregular sleep | 9/858 | **13.3 (1.80-98.5)** | 28/858 | 0.70 (0.35-1.40) | 26/858 | 1.93 (0.83-4.51) | 37/858 | 0.92 (0.48-1.74) | 3/858 | 0.26 (0.04-1.47) | 19/858 | 1.02 (0.41-2.53) |

All models were adjusted for age, sex, race, educational level, marital status, family income, body mass index, alcohol intake, smoking status, alternative healthy eating index, total energy intake, diabetes, cardiovascular disease, cancer, hypertension, hyperlipidaemia, accelerometer-measured physical activity volume and sleep duration.

## **Supplementary Table 3.** Sensitivity analysis of the associations of diurnal patterns of physical activity and sleep with all-cause mortality by including self-reported amount of moderate and vigorous physical activity, sedentary time, and sleep duration at night as covariates.

|  | **Case/N** | **Model 1** | **Model 2** | **Model 3** |
| --- | --- | --- | --- | --- |
| **Physical activity** |  |  |  |  |
| Early-morning | 242/2159 | **1.30 (1.06-1.59)** | **1.33 (1.11-1.60)** | **1.30 (1.08-1.58)** |
| Late-afternoon | 112/1677 | 1.44 (0.97-2.15) | 1.19 (0.84-1.69) | 1.21 (0.86-1.71) |
| Midday | 235/2837 | Ref | Ref | Ref |
| **Sleep** |  |  |  |  |
| Irregular sleep | 292/2493 | **1.78 (1.36-2.33)** | **1.44 (1.09-1.90)** | **1.32 (1.00-1.73)** |
| Morning lark | 193/2245 | Ref | Ref | Ref |
| Night owl | 104/1935 | 1.06 (0.68-1.65) | 1.01 (0.64-1.58) | 0.95 (0.60-1.50) |
| **Joint pattern of physical activity and sleep** | | | | |
| Early-morning & Morning lark | 109/1146 | 1.04 (0.77-1.40) | 1.09 (0.80-1.47) | 1.04 (0.81-1.35) |
| Early-morning & Night owl | 13/246 | 1.11 (0.46-2.71) | 1.17 (0.47-2.92) | 1.06 (0.46-2.45) |
| Early-morning & Irregular sleep | 120/767 | **2.32 (1.65-3.25)** | **1.91 (1.37-2.67)** | **1.70 (1.22-2.37)** |
| Late-afternoon & Morning lark | 3/59 | 2.75 (0.93-8.16) | 2.31 (0.83-6.41) | 2.00 (0.74-5.41) |
| Late-afternoon & Night owl | 31/750 | 1.25 (0.75-2.09) | 0.97 (0.56-1.68) | 0.93 (0.52-1.65) |
| Late-afternoon & Irregular sleep | 78/868 | 2.29 (1.43-3.65) | 1.57 (0.98-2.53) | 1.45 (0.94-2.24) |
| Midday & Morning lark | 81/1040 | Ref | Ref | Ref |
| Midday & Night owl | 60/939 | 1.02 (0.63-1.66) | 0.98 (0.58-1.63) | 0.91 (0.54-1.52) |
| Midday & Irregular sleep | 94/858 | 1.38 (0.94-2.02) | 1.14 (0.75-1.73) | 1.02 (0.70-1.49) |

Model 1: adjusted for age and sex;

Model 2: additionally adjusted for race, educational level, marital status, family income, body mass index, alcohol intake, smoking status, alternative healthy eating index, diabetes, cardiovascular disease, cancer, hypertension, and hyperlipidemia based on model 1.

Model 3: additionally adjusted for self-reported amount of moderate and vigorous physical activity, sedentary time, and sleep duration at night as covariates based on model 2.

## **Supplementary Table 4.** Sensitivity analysis of the associations of diurnal patterns of physical activity and sleep with all-cause mortality by excluding participants with diabetes or CVD at baseline (N=5329).

|  | **Case/N** | **Model 1** | **Model 2** | **Model 3** |
| --- | --- | --- | --- | --- |
| **Physical activity** |  |  |  |  |
| Early-morning | 109/1667 | 1.21 (0.87-1.68) | 1.20 (0.85-1.68) | 1.23 (0.86-1.77) |
| Late-afternoon | 61/1394 | 1.50 (0.96-2.35) | 1.17 (0.71-1.92) | 1.15 (0.68-1.96) |
| Midday | 123/2268 | Ref | Ref | Ref |
| **Sleep** |  |  |  |  |
| Irregular sleep | 127/1883 | 1.69 (1.11-2.55) | 1.45 (0.98-2.14) | 1.60 (0.98-2.61) |
| Morning lark | 109/1844 | Ref | Ref | Ref |
| Night owl | 57/1602 | 1.15 (0.64-2.08) | 1.14 (0.61-2.10) | 1.15 (0.62-2.14) |
| **Joint pattern of physical activity and sleep** | | | | |
| Early-morning & Morning lark | 59/922 | 1.02 (0.72-1.44) | 1.02 (0.70-1.48) | 1.03 (0.70-1.51) |
| Early-morning & Night owl | 7/212 | 1.50 (0.48-4.69) | 1.76 (0.56-5.51) | 1.88 (0.57-6.20) |
| Early-morning & Irregular sleep | 43/533 | 2.04 (1.20-3.48) | 1.68 (1.04-2.72) | 1.94 (1.12-3.36) |
| Late-afternoon & Morning lark | 2/53 | 2.64 (0.47-14.7) | 2.34 (0.37-14.8) | 2.37 (0.39-14.3) |
| Late-afternoon & Night owl | 18/632 | 1.51 (0.74-3.10) | 1.08 (0.51-2.28) | 1.09 (0.51-2.32) |
| Late-afternoon & Irregular sleep | 41/709 | 2.30 (1.43-3.70) | 1.61 (0.89-2.94) | 1.75 (0.96-3.21) |
| Midday & Morning lark | 48/869 | Ref | Ref | Ref |
| Midday & Night owl | 32/758 | 1.05 (0.53-2.06) | 1.03 (0.51-2.10) | 1.02 (0.50-2.07) |
| Midday & Irregular sleep | 43/641 | 1.45 (0.77-2.74) | 1.27 (0.63-2.56) | 1.41 (0.68-2.93) |

Model 1: adjusted for age and sex;

Model 2: additionally adjusted for race, educational level, marital status, family income, body mass index, alcohol intake, smoking status, alternative healthy eating index, diabetes, cardiovascular disease, cancer, hypertension, and hyperlipidemia based on model 1.

Model 3: additionally adjusted for accelerometer-measured physical activity volume and sleep duration per day based on model 2.

## **Supplementary Table 5.** Sensitivity analysis of the associations of diurnal patterns of physical activity and sleep with all-cause mortality by excluding participants with diabetes, CVD, cancer, hypertension or hyperlipidemia at baseline (N=2819).

|  | **Case/N** | **Model 1** | **Model 2** | **Model 3** |
| --- | --- | --- | --- | --- |
| **Physical activity** |  |  |  |  |
| Early-morning | 19/750 | 1.03 (0.54-1.97) | 1.16 (0.53-2.56) | 1.17 (0.54-2.50) |
| Late-afternoon | 22/905 | 1.61 (0.78-3.32) | 1.39 (0.64-3.01) | 1.19 (0.52-2.71) |
| Midday | 30/1164 | Ref | Ref | Ref |
| **Sleep** |  |  |  |  |
| Irregular sleep | 34/1005 | 2.01 (0.85-4.73) | 1.83 (0.78-4.30) | 4.11 (1.03-16.4) |
| Morning lark | 21/871 | Ref | Ref | Ref |
| Night owl | 16/943 | 1.20 (0.39-3.68) | 1.16 (0.39-3.47) | 1.35 (0.43-4.29) |
| **Joint pattern of physical activity and sleep** | | | | |
| Early-morning & Morning lark | 9/420 | 0.67 (0.23-1.93) | 0.66 (0.21-2.07) | 0.68 (0.22-2.08) |
| Early-morning & Night owl | 3/101 | 2.07 (0.33-13.1) | 2.28 (0.35-14.7) | 4.27 (0.77-23.5) |
| Early-morning & Irregular sleep | 7/229 | 2.03 (0.84-4.89) | 2.10 (0.99-4.45) | 4.78 (1.38-16.6) |
| Late-afternoon & Morning lark | 1/32 | 4.55 (0.53-39.3) | 4.52 (0.46-44.7) | 5.19 (0.57-47.5) |
| Late-afternoon & Night owl | 5/424 | 1.76 (0.50-6.22) | 1.31 (0.36-4.70) | 1.38 (0.35-5.41) |
| Late-afternoon & Irregular sleep | 16/449 | 2.50 (1.12-5.54) | 1.80 (0.58-5.54) | 3.74 (0.84-16.7) |
| Midday & Morning lark | 11/419 | Ref | Ref | Ref |
| Midday & Night owl | 8/418 | 0.78 (0.24-2.54) | 0.73 (0.20-2.72) | 0.81 (0.21-3.16) |
| Midday & Irregular sleep | 11/327 | 1.86 (0.54-6.40) | 1.48 (0.36-6.18) | 4.08 (0.52-31.9) |

Model 1: adjusted for age and sex;

Model 2: additionally adjusted for race, educational level, marital status, family income, body mass index, alcohol intake, smoking status, alternative healthy eating index, diabetes, cardiovascular disease, cancer, hypertension, and hyperlipidemia based on model 1.

Model 3: additionally adjusted for accelerometer-measured physical activity volume and sleep duration per day based on model 2.

## **Supplementary Table 6.** Sensitivity analysis of the associations of diurnal patterns of physical activity and sleep with all-cause mortality by excluding participants who reported no moderate-to-vigorous physical activity (n=6657).

|  | **Case/N** | **Model 1** | **Model 2** | **Model 3** |
| --- | --- | --- | --- | --- |
| **Physical activity** |  |  |  |  |
| Early-morning | 239/2153 | **1.29 (1.05-1.58)** | **1.33 (1.11-1.59)** | **1.36 (1.13-1.63)** |
| Late-afternoon | 112/1674 | 1.45 (0.97-2.15) | 1.20 (0.85-1.70) | 1.21 (0.86-1.71) |
| Midday | 234/2830 | Ref | Ref | Ref |
| **Sleep** |  |  |  |  |
| Irregular sleep | 290/2486 | **1.77 (1.35-2.33)** | **1.44 (1.09-1.91)** | **1.42 (1.01-2.01)** |
| Morning lark | 192/2240 | Ref | Ref | Ref |
| Night owl | 103/1931 | 1.05 (0.67-1.65) | 1.00 (0.64-1.58) | 1.02 (0.65-1.60) |
| **Joint pattern of physical activity and sleep** | | | | |
| Early-morning & Morning lark | 108/1144 | 1.03 (0.77-1.39) | 1.08 (0.80-1.46) | 1.10 (0.81-1.50) |
| Early-morning & Night owl | 13/146 | 1.11 (0.45-2.70) | 1.17 (0.47-2.92) | 1.23 (0.49-3.10) |
| Early-morning & Irregular sleep | 18/763 | **2.29 (1.63-3.23)** | **1.90 (1.36-2.67)** | **1.92 (1.32-2.79)** |
| Late-afternoon & Morning lark | 3/59 | 2.74 (0.92-8.11) | 2.30 (0.83-6.40) | 2.48 (0.90-6.84) |
| Late-afternoon & Night owl | 31/749 | 1.25 (0.75-2.08) | 0.97 (0.56-1.67) | 1.00 (0.58-1.73) |
| Late-afternoon & Irregular sleep | 78/866 | 2.28 (1.43-3.63) | 1.57 (0.98-2.53) | 1.56 (0.94-2.58) |
| Midday & Morning lark | 81/1037 | Ref | Ref | Ref |
| Midday & Night owl | 59/936 | 1.01 (0.62-1.64) | 0.97 (0.57-1.63) | 0.98 (0.58-1.64) |
| Midday & Irregular sleep | 94/857 | 1.37 (0.94-2.01) | 1.14 (0.75-1.73) | 1.13 (0.74-1.71) |

Model 1: adjusted for age and sex;

Model 2: additionally adjusted for race, educational level, marital status, family income, body mass index, alcohol intake, smoking status, alternative healthy eating index, diabetes, cardiovascular disease, cancer, hypertension, and hyperlipidemia based on model 1.

Model 3: additionally adjusted for accelerometer-measured physical activity volume and sleep duration perday based on model 2.

## **Supplementary Table 7.** Sensitivity analysis of the associations of diurnal patterns of physical activity and sleep with all-cause mortality by excluding participants with abnormal (<1% or >99%) accelerometer-measured physical activity volume or sleep duration (n=6425).

|  | **Case/N** | **Model 1** | **Model 2** | **Model 3** |
| --- | --- | --- | --- | --- |
| **Physical activity** |  |  |  |  |
| Early-morning | 225/2079 | 1.24 (1.00-1.54) | 1.29 (1.06-1.57) | 1.32 (1.08-1.61) |
| Late-afternoon | 105/1609 | 1.39 (0.93-2.09) | 1.17 (0.82-1.67) | 1.18 (0.82-1.69) |
| Midday | 222/2737 | Ref | Ref | Ref |
| **Sleep** |  |  |  |  |
| Irregular sleep | 259/2320 | 1.70 (1.29-2.24) | 1.37 (1.03-1.81) | 1.40 (1.00-1.98) |
| Morning lark | 191/2204 | Ref | Ref | Ref |
| Night owl | 102/1901 | 1.05 (0.68-1.63) | 1.00 (0.65-1.55) | 1.02 (0.66-1.58) |
| **Joint pattern of physical activity and sleep** | | | | |
| Early-morning & Morning lark | 108/1123 | 1.04 (0.77-1.40) | 1.09 (0.80-1.47) | 1.11 (0.82-1.50) |
| Early-morning & Night owl | 13/242 | 1.11 (0.46-2.70) | 1.17 (0.47-2.88) | 1.24 (0.50-3.06) |
| Early-morning & Irregular sleep | 104/714 | 2.15 (1.49-3.10) | 1.79 (1.25-2.56) | 1.86 (1.25-2.77) |
| Late-afternoon & Morning lark | 3/57 | 2.76 (0.92-8.28) | 2.38 (0.86-6.54) | 2.54 (0.93-6.94) |
| Late-afternoon & Night owl | 29/734 | 1.21 (0.72-2.03) | 0.96 (0.56-1.65) | 0.98 (0.57-1.70) |
| Late-afternoon & Irregular sleep | 73/818 | 2.16 (1.35-3.46) | 1.50 (0.93-2.41) | 1.53 (0.93-2.52) |
| Midday & Morning lark | 80/1024 | Ref | Ref | Ref |
| Midday & Night owl | 60/925 | 1.03 (0.64-1.67) | 0.99 (0.60-1.64) | 1.00 (0.60-1.65) |
| Midday & Irregular sleep | 82/788 | 1.36 (0.92-2.02) | 1.11 (0.73-1.70) | 1.14 (0.75-1.74) |

Model 1: adjusted for age and sex;

Model 2: additionally adjusted for race, educational level, marital status, family income, body mass index, alcohol intake, smoking status, alternative healthy eating index, diabetes, cardiovascular disease, cancer, hypertension, and hyperlipidemia based on model 1.

Model 3: additionally adjusted for accelerometer-measured physical activity volume and sleep duration perday based on model 2.

## **Supplementary Table 8.** Sensitivity analysis of the associations of diurnal patterns of physical activity and sleep with all-cause mortality by excluding participants with less than 2 years follow-up (n=6,566).

|  | **Case/N** | **Model 1** | **Model 2** | **Model 3** |
| --- | --- | --- | --- | --- |
| **Physical activity** |  |  |  |  |
| Early-morning | 198/2115 | 1.28 (1.01-1.64) | 1.34 (1.07-1.67) | 1.37 (1.10-1.70) |
| Late-afternoon | 90/1655 | 0.54 (0.98-2.41) | 1.27 (0.84-1.91) | 1.27 (0.85-1.91) |
| Midday | 194/2796 | Ref | Ref | Ref |
| **Sleep** |  |  |  |  |
| Irregular sleep | 237/2438 | 1.73 (1.23-2.43) | 1.37 (0.99-1.90) | 1.37 (1.10-1.70) |
| Morning lark | 161/2213 | Ref | Ref | Ref |
| Night owl | 84/1915 | 1.00 (0.60-1.65) | 0.93 (0.57-1.54) | 1.27 (0.85-1.91) |
| **Joint pattern of physical activity and sleep** | | | | |
| Early-morning & Morning lark | 88/1125 | 1.04 (0.70-1.54) | 1.11 (0.75-1.65) | 1.14 (0.76-1.71) |
| Early-morning & Night owl | 9/242 | 0.88 (0.34-2.32) | 0.96 (0.38-2.43) | 1.03 (0.40-2.67) |
| Early-morning & Irregular sleep | 101/748 | 2.30 (1.48-3.58) | 1.89 (0.23-2.90) | 1.97 (1.30-2.99) |
| Late-afternoon & Morning lark | 3/59 | 3.43 (1.20-9.81) | 2.63 (0.97-7.10) | 2.79 (1.03-7.58) |
| Late-afternoon & Night owl | 28/747 | 1.35 (0.79-2.33) | 1.03 (0.58-1.81) | 1.05 (0.60-1.86) |
| Late-afternoon & Irregular sleep | 59/849 | 2.28 (1.36-3.83) | 1.56 (0.91-2.66) | 1.60 (0.95-2.71) |
| Midday & Morning lark | 70/1029 | Ref | Ref | Ref |
| Midday & Night owl | 47/926 | 0.99 (0.62-1.57) | 0.93 (0.55-1.55) | 0.95 (0.57-1.58) |
| Midday & Irregular sleep | 77/841 | 1.36 (0.90-2.04) | 1.11 (0.70-1.76) | 1.14 (0.77-1.69) |

Model 1: adjusted for age and sex;

Model 2: additionally adjusted for race, educational level, marital status, family income, body mass index, alcohol intake, smoking status, alternative healthy eating index, diabetes, cardiovascular disease, cancer, hypertension, and hyperlipidemia based on model 1.

Model 3: additionally adjusted for accelerometer-measured physical activity volume and sleep duration perday based on model 2.

## **Supplementary Table 9.** Sensitivity analysis of the associations of diurnal patterns of physical activity and sleep with all-cause mortality by imputing missing covariates (n=8,849).

|  | **Case/N** | **Model 1** | **Model 2** | **Model 3** |
| --- | --- | --- | --- | --- |
| **Physical activity** |  |  |  |  |
| Early-morning | 341/2894 | 1.13 (0.98-1.31) | 1.11 (0.96-1.29) | 1.11 (0.95-1.29) |
| Late-afternoon | 154/2148 | 1.12 (0.86-1.47) | 1.00 (0.82-1.22) | 1.00 (0.82-1.22) |
| Midday | 357/3807 | 1.00 (Reference) | 1.00 (Reference) | 1.00 (Reference) |
| **Sleep** |  |  |  |  |
| Irregular sleep | 436/3350 | 1.83 (1.45-2.31) | 1.43 (1.12-1.82) | 1.43 (1.12-1.82) |
| Morning lark | 262/2913 | 1.00 (Reference) | 1.00 (Reference) | 1.00 (Reference) |
| Night owl | 154/2586 | 0.99 (0.71-1.37) | 0.91 (0.65-1.27) | 0.91 (0.65-1.27) |
| **Joint pattern of physical activity and sleep** | | | | |
| Early-morning & Morning lark | 720/7665 | 0.93 (0.68-1.27) | 0.91 (0.67-1.23) | 0.91 (0.67-1.22) |
| Early-morning & Night owl | 110/1685 | 1.03 (0.53-2.01) | 0.94 (0.50-1.79) | 0.94 (0.50-1.79) |
| Early-morning & Irregular sleep | 875/5120 | 2.00 (1.50-2.66) | 1.53 (1.14-2.06) | 1.53 (1.14-2.06) |
| Late-afternoon & Morning lark | 10/345 | 1.06 (0.30-3.83) | 0.98 (0.30-3.28) | 0.99 (0.30-3.31) |
| Late-afternoon & Night owl | 205/4675 | 0.79 (0.51-1.22) | 0.62 (0.39-0.98) | 0.62 (0.39-1.00) |
| Late-afternoon & Irregular sleep | 555/5720 | 1.99 (1.37-2.89) | 1.41 (0.99-2.00) | 1.41 (1.00-2.00) |
| Midday & Morning lark | 580/6555 | 1.00 (Reference) | 1.00 (Reference) | 1.00 (Reference) |
| Midday & Night owl | 455/6570 | 0.96 (0.68-1.35) | 0.89 (0.62-1.28) | 0.89 (0.62-1.28) |
| Midday & Irregular sleep | 750/5910 | 1.43 (1.00-2.05) | 1.10 (0.77-1.57) | 1.10 (0.77-1.57) |

Model 1: adjusted for age and sex;

Model 2: additionally adjusted for race, educational level, marital status, family income, body mass index, alcohol intake, smoking status, alternative healthy eating index, diabetes, cardiovascular disease, cancer, hypertension, and hyperlipidemia based on model 1.

Model 3: additionally adjusted for accelerometer-measured physical activity volume and sleep duration perday based on model 2.

# Supplementary Figures

**
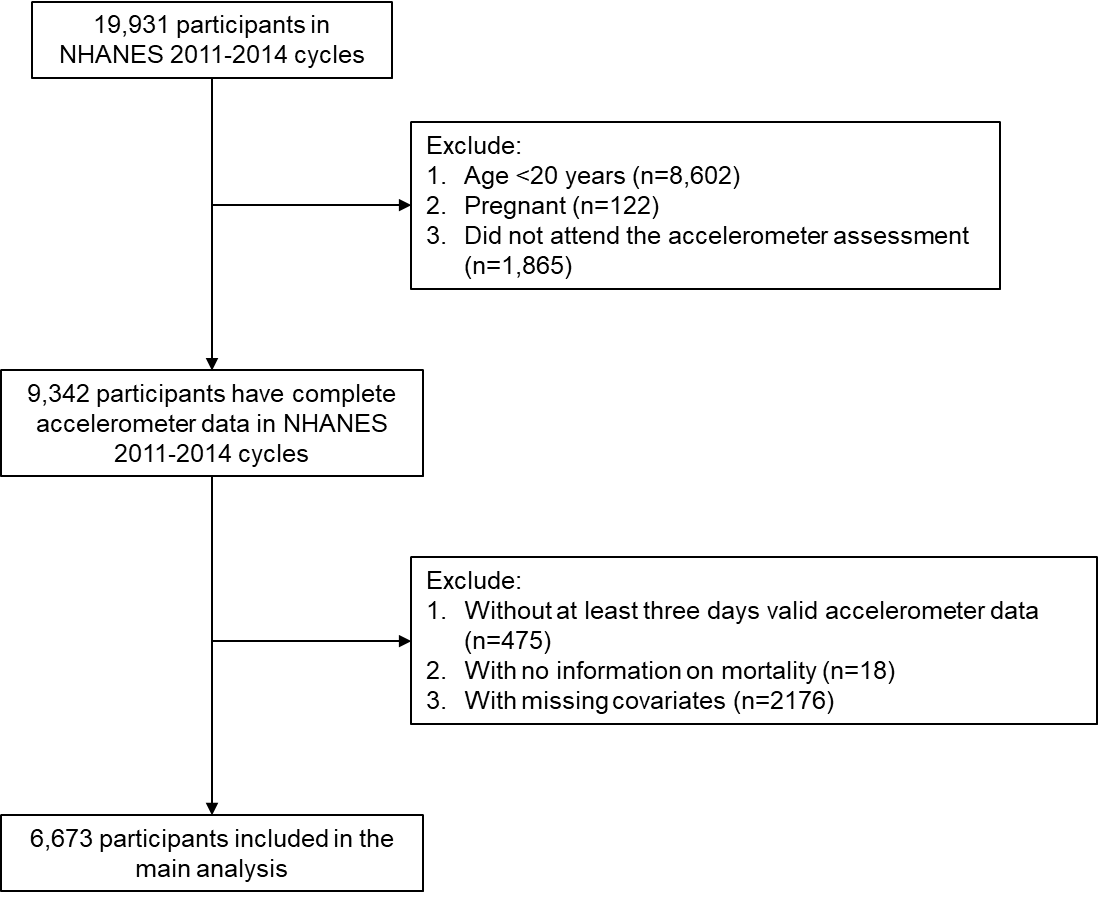
**

## **Supplementary Figure 1.** Flow chart of the selection of study population.


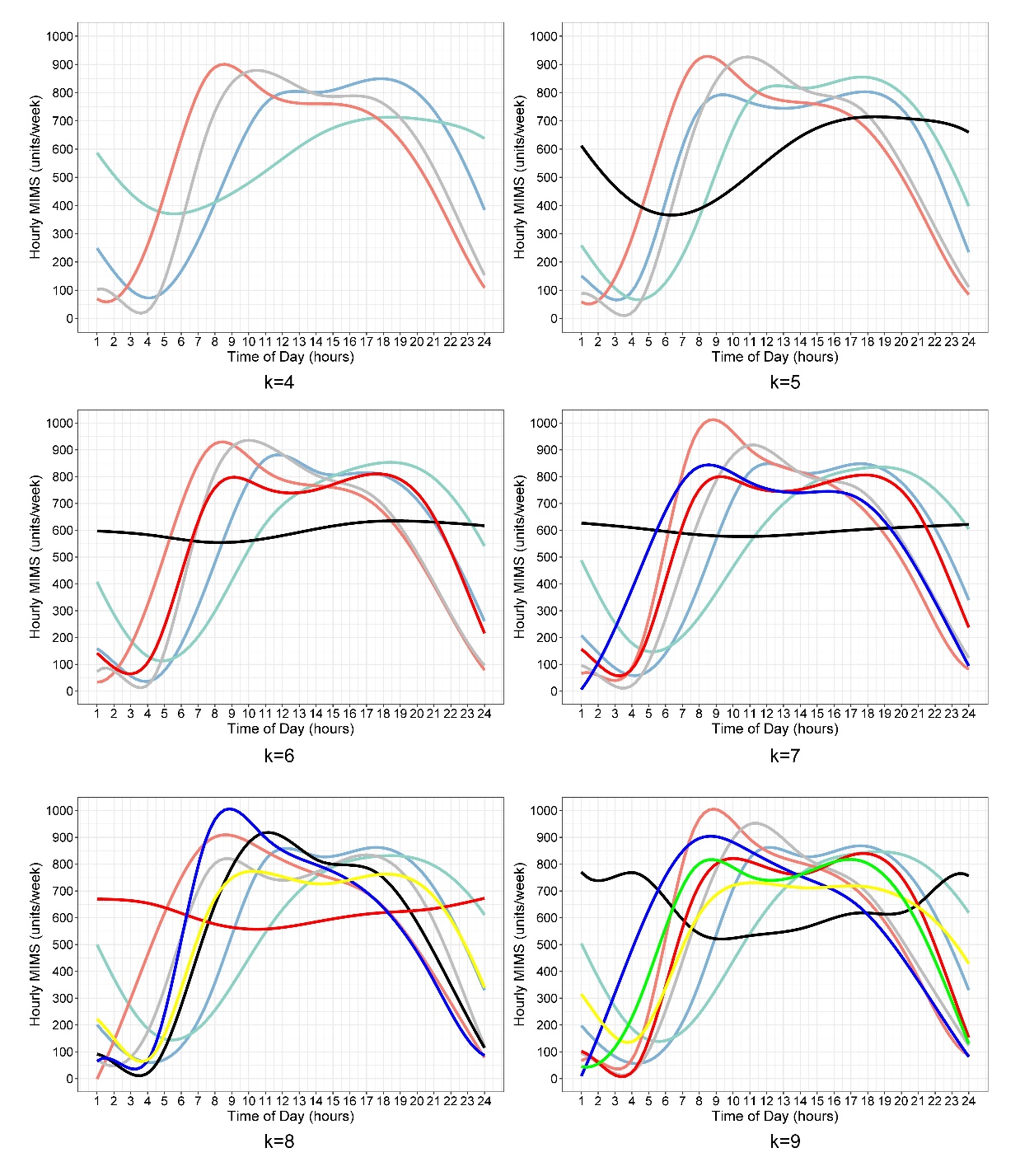


## **Supplementary Figure 2.** Diurnal patterns of physical activity by machine learning-based clustering using a higher number of clusters (k=4~9).


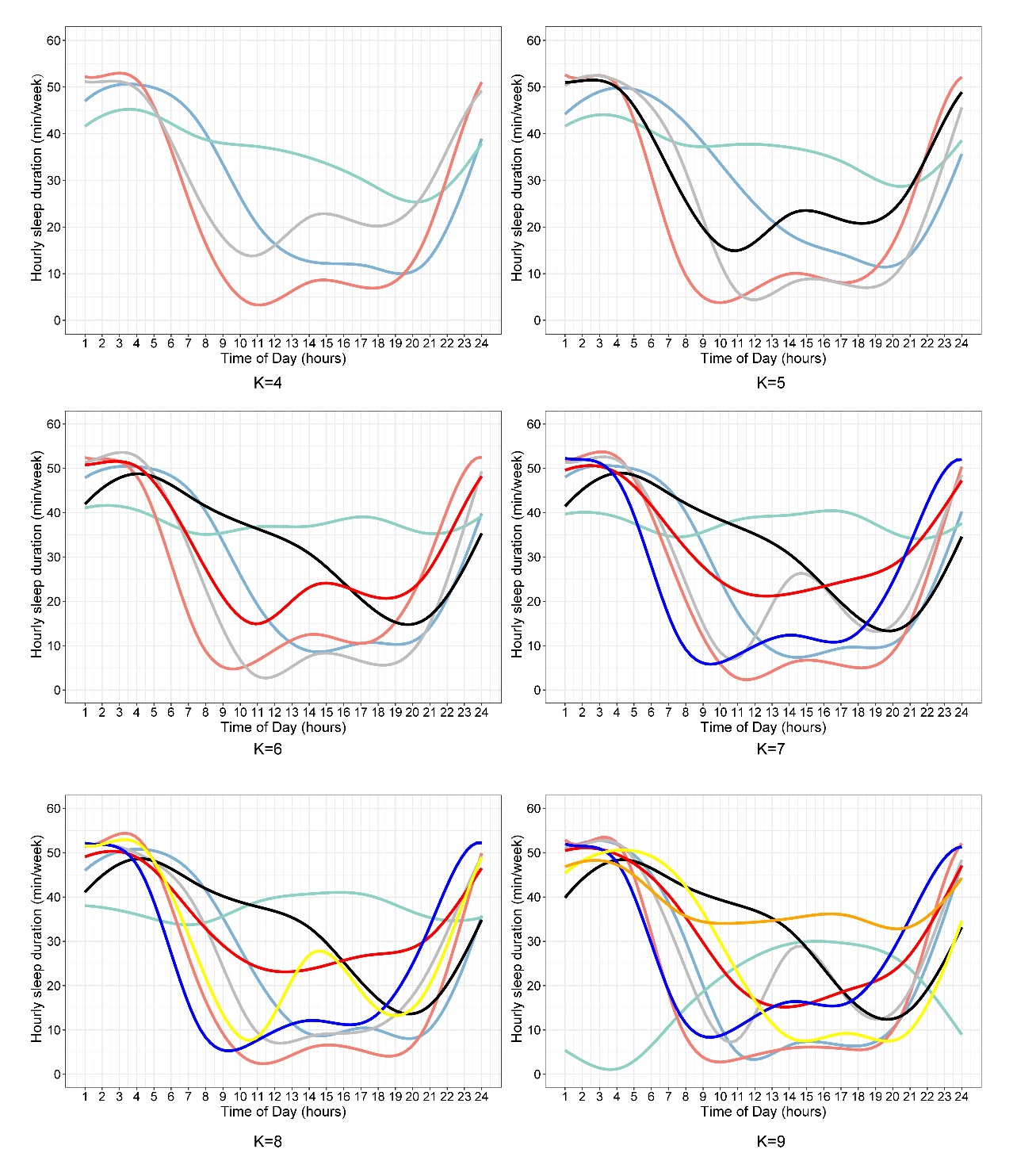


## **Supplementary Figure 3.** Diurnal patterns of sleep by machine learning-based clustering using a higher number of clusters (k=4~9).

##
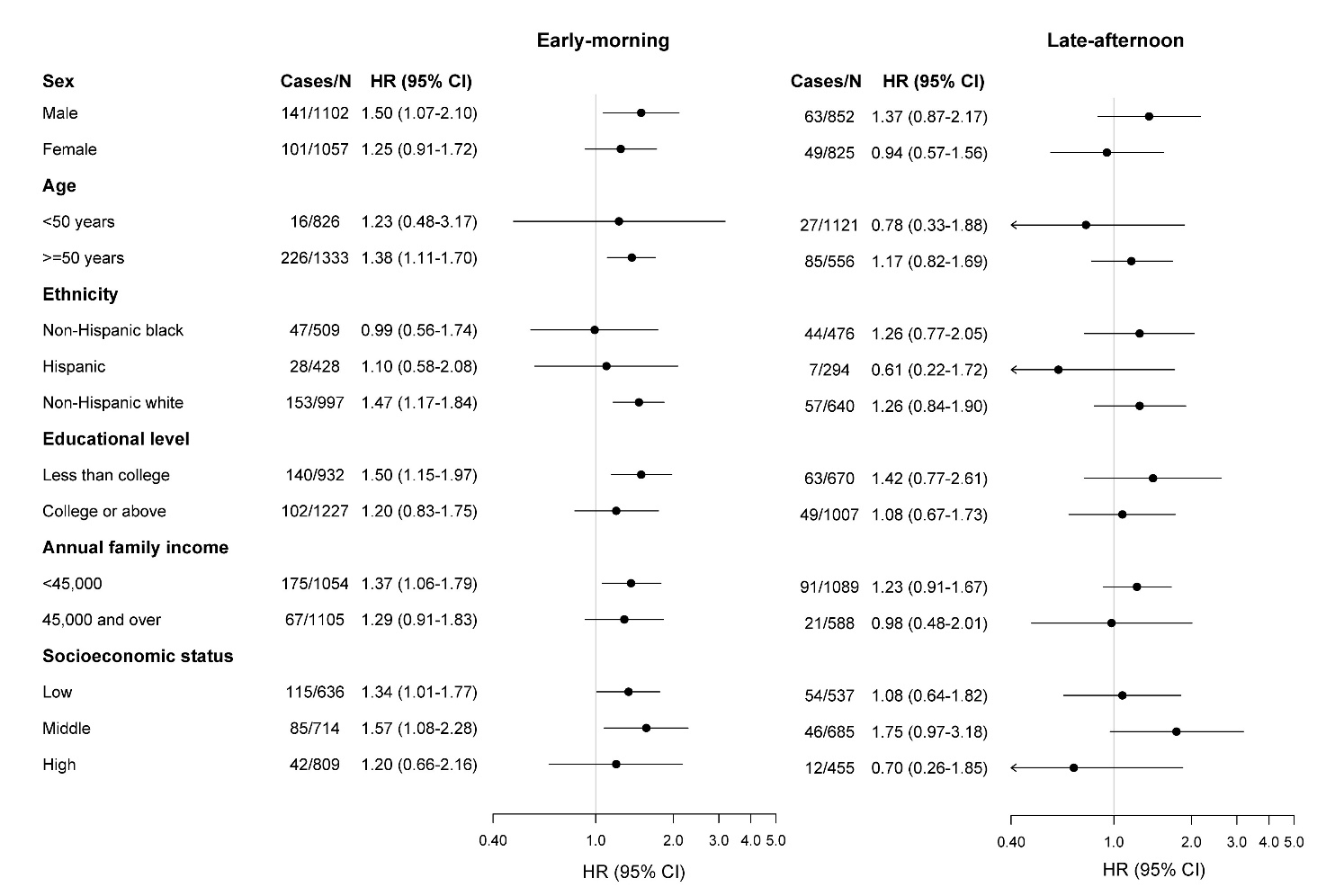
**Supplementary Figure 4.** Subgroup analysis for the associations of diurnal patterns of physical activity with all-cause mortality.


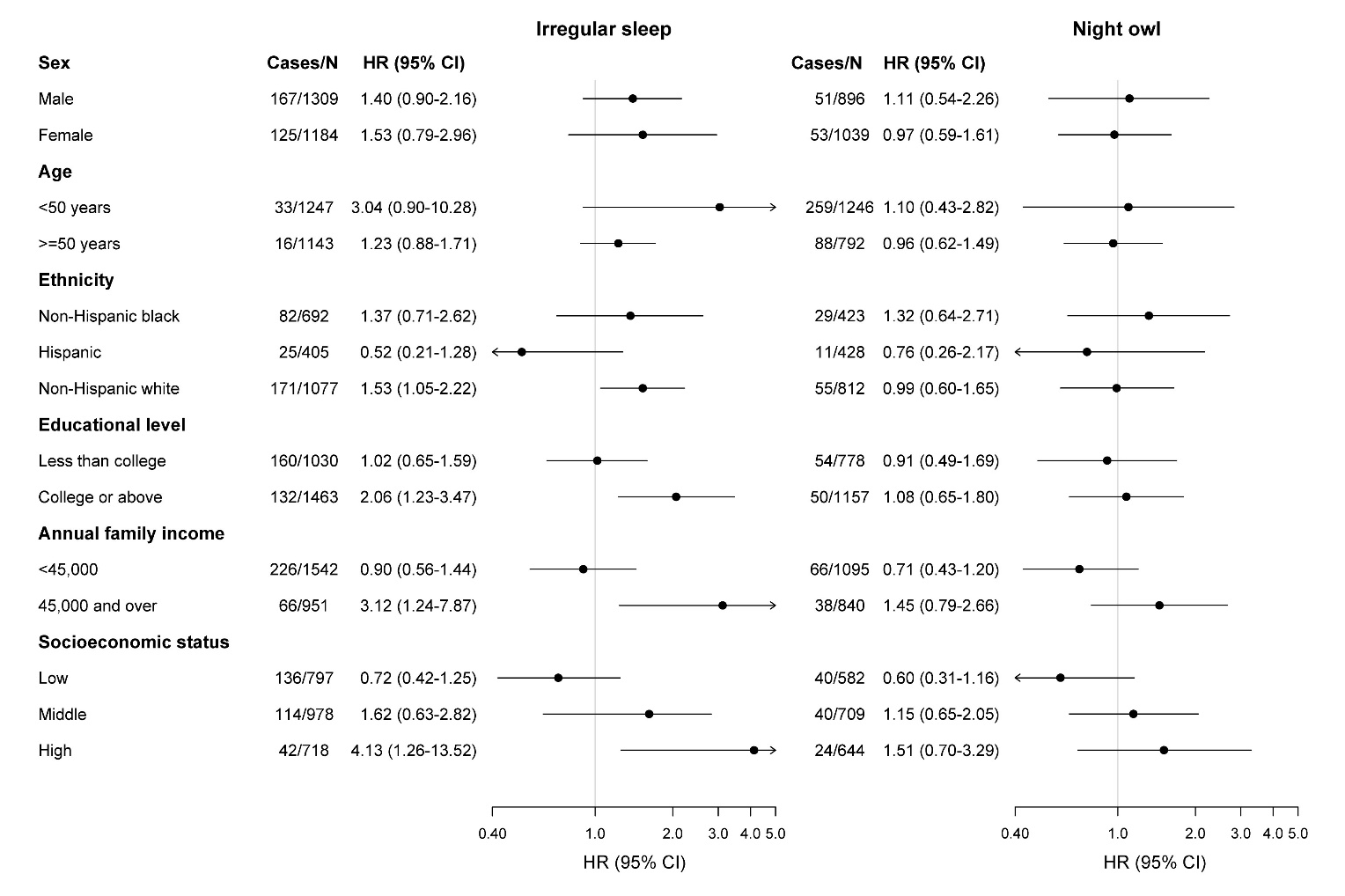


## **Supplementary Figure 5.** Subgroup analysis for the associations of diurnal patterns of sleep with all-cause mortality


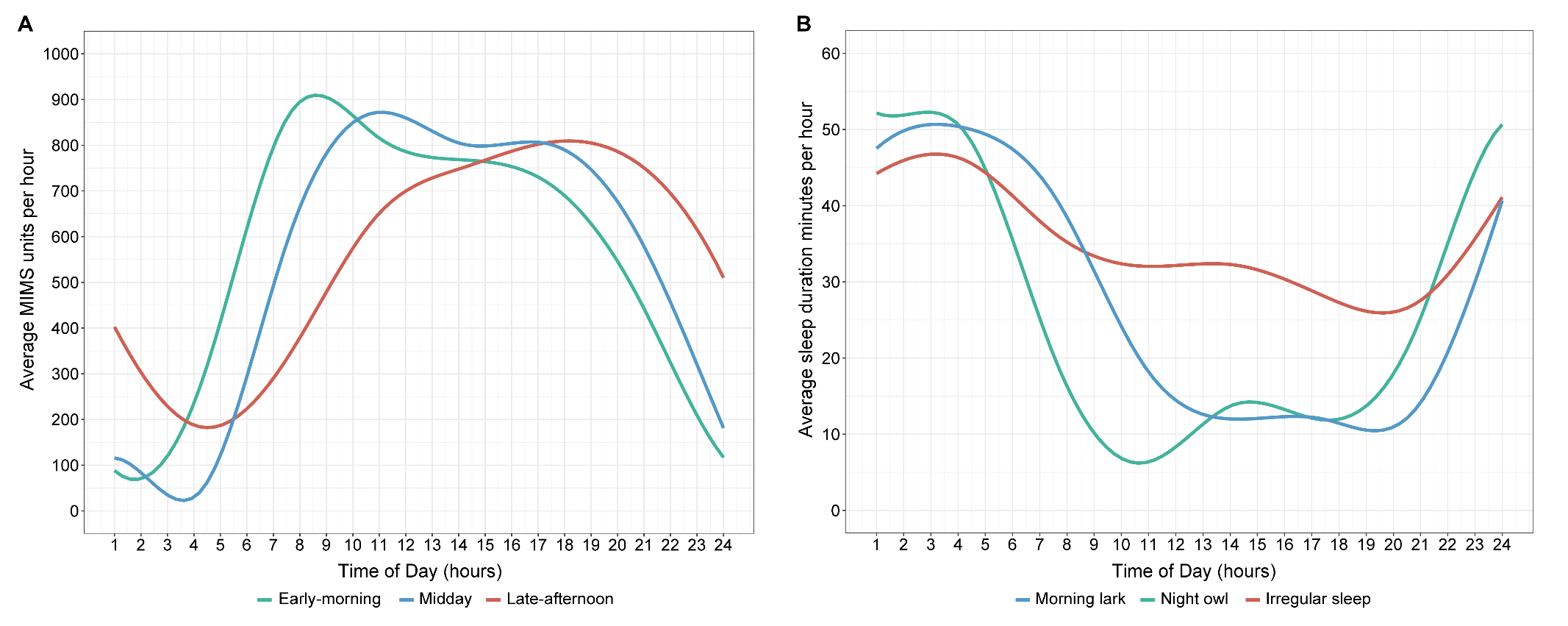


## **Supplementary Figure 6.** Sensitivity analysis of diurnal patterns of physical activity (A) and sleep (B) by including participants with missing covariates.

A. Diurnal pattern of physical activity. Hourly monitor-independent movement summary (MIMS) units were calculated as the MIMS accumulated in each hour of the day. The average MIMS accumulated per hour across all subjects in each cluster was shown in different colours.

B. Diurnal pattern of sleep. Hourly sleep duration was calculated as the sleep duration accumulated in each hour of the day. The average sleep duration accumulated per hour across all subjects in each cluster was shown in different colours.
